# Synergistic advancements in sewage-driven microbial fuel cells: novel carbon nanotube cathodes and biomass-derived anodes for efficient renewable energy generation and wastewater treatment

**DOI:** 10.3389/fchem.2023.1286572

**Published:** 2023-11-24

**Authors:** Nasser A. M. Barakat, Shimaa Gamal, Hak Yong Kim, Nasser M. Abd El-Salam, Hassan Fouad, Olfat A. Fadali, Hager M. Moustafa, Omina H. Abdelraheem

**Affiliations:** ^1^ Chemical Engineering Department, Faculty of Engineering, Minia University, El-Minia, Egypt; ^2^ Department of Nano Convergence Engineering, Jeonbuk National University, Jeonju, Republic of Korea; ^3^ Department of Organic Materials and Fiber Engineering, Jeonbuk National University, Jeonju, Republic of Korea; ^4^ Natural Science Department, Community College, King Saud University, Riyadh, Saudi Arabia; ^5^ Biomedical Engineering Department, Faculty of Engineering, Helwan University, Helwan, Egypt; ^6^ Sciences Engineering Department, Faculty of Engineering, Beni-Suef University, Beni-Suef, Egypt

**Keywords:** microbial fuel cells, electrode design, 3D anodes, renewable energy, activated carbon-carbon nanotube composite, sewage water

## Abstract

Microbial fuel cells (MFCs) offer a dual solution of generating electrical energy from organic pollutants-laden wastewater while treating it. This study focuses on enhancing MFC performance through innovative electrode design. Three-dimensional (3D) anodes, created from corncobs and mango seeds via controlled graphitization, achieved remarkable power densities. The newly developed electrode configurations were evaluated within sewage wastewater-driven MFCs without the introduction of external microorganisms or prior treatment of the wastewater. At 1,000°C and 1,100°C graphitization temperatures, corncob and mango seed anodes produced 1,963 and 2,171 mW/m^2^, respectively, nearly 20 times higher than conventional carbon cloth and paper anodes. An advanced cathode composed of an activated carbon-carbon nanotube composite was introduced, rivaling expensive platinum-based cathodes. By optimizing the thermal treatment temperature and carbon nanotube content of the proposed cathode, comparable or superior performance to standard Pt/C commercial cathodes was achieved. Specifically, MFCs assembled with corncob anode with the proposed and standard Pt/C cathodes reached power densities of 1,963.1 and 2,178.6 mW/m^2^, respectively. Similarly, when utilizing graphitized mango seeds at 1,100°C, power densities of 2,171 and 2,151 mW/m^2^ were achieved for the new and standard cathodes, respectively. Furthermore, in continuous operation with a flow rate of 2 L/h, impressive chemical oxygen demand (COD) removal rates of 77% and 85% were achieved with corncob and mango seed anodes, respectively. This work highlights the significance of electrode design for enhancing MFC efficiency in electricity generation and wastewater treatment.

## 1 Introduction

The pursuit of alternative renewable energy sources has gained paramount importance in contemporary scientific research, addressing global challenges such as climate change and fossil fuel depletion (Slate et al., 2019; Kugarajah and Dharmalingam, 2020; Ma et al., 2023). In this context, microbial fuel cells (MFCs) have emerged as a noteworthy solution with the potential to contribute significantly to both sustainable energy generation and environmental remediation. MFCs represent an innovative class of fuel cells that operate under mild conditions and use cost-effective substrates, enabling the efficient conversion of renewable biomass and organic wastewater into bioelectricity through a biooxidation process (Mouhib et al., 2019; Nguyen and Taguchi, 2020; Gupta et al., 2021; Liu et al., 2021).

The fundamental operating principle of MFCs is rooted in harnessing the metabolic activities of microorganisms residing within the anode chamber. Here, organic matter and biomass undergo biocatalysis by active bacteria, yielding protons and electrons as metabolic byproducts. Protons migrate across a proton-exchange membrane (PEM) to the cathode, while electrons engage in extracellular electron transfer (EET) processes (Du et al., 2019; Guo et al., 2019; Marks et al., 2019). The distinguishing feature of MFCs lies in their utilization of active microorganisms as biocatalysts in the anode, contrasting traditional fuel cells that use noble metals. This convergence of biocatalysis and electrocatalysis bestows several advantages, including mild reaction conditions, broad substrate compatibility, high activity, selectivity, and the potential for sustainable energy conversion (Chen et al., 2020). Despite these merits, practical application of MFCs faces challenges such as high capital expenditure, low power density, sluggish EET efficiency, and limited stability (Baca et al., 2016; Liu et al., 2019). The power output density of MFCs is influenced by diverse factors, ranging from cell configurations to anodic and cathodic materials (Mohan and Das, 2009; Awan et al., 2014; Ren et al., 2022). To address these limitations, significant efforts have been dedicated to enhancing power density through strategies like the integration of external electron mediators, optimizing cell configurations, selecting appropriate fuels, and developing advanced electrode materials (Mohan and Das, 2009; Mehdinia et al., 2014; Gao et al., 2021).

Strategies to modify anodic substrate materials, which are the most effective parameter enhancing the MFCs performance, have been harnessed to reduce anodic activation overpotential, enhance EET capabilities, and elevate power generation performance. Studies have demonstrated the efficacy of ammonia-treated graphite brushes, ammonia-treated carbon cloth, and modified carbon cloth bioanodes, in promoting electrogenic bacteria attachment and accelerating EET rates (Logan et al., 2007; Li et al., 2014). Acidic modification of carbon cloth bioanodes further amplified the accessible surface areas, reduced anode resistance, and facilitated biofilm formation, resulting in accelerated MFC startup times and improved power density production (Li et al., 2014). Phenolic carbon felt (PCF) modifications have been explored through carbonization processes at varying temperatures, demonstrating that PCF-900, characterized by higher surface areas and favorable hydrophilic properties, exhibited optimal adhesion of active bacteria and highest power density production (Zhang, 2021a). Despite these advancements, inherent limitations of anode substrate materials, such as inadequate biocompatibility, electrocatalytic activity, and electron transfer abilities, persist (Logan et al., 2007). To address these challenges, research organizations have sought to fabricate anode modification materials that offer biocompatibility, macroporous structures, electroconductivity, electroactive areas, and chemical stability (Niessen et al., 2004; Santoro et al., 2016). The endeavor to synthesize such materials with ease, environmental friendliness, cost-effectiveness, and robust electrical conductivity is ongoing.

In recent years, the utilization of biomass-derived carbon nanomaterials with distinct natural structures has garnered significant attention for their integration into anodic modification materials within MFCs. These novel biomass materials have been harnessed for their unique advantages, including cost-effective production, eco-friendly sourcing from nature, wide availability, and remarkable biological compatibility. As exemplified by the work of Yuan et al. (Yuan et al., 2013), the high-temperature carbonization of natural reticulated loofah sponges and polyaniline (PANI) yielded nitrogen-doped macroporous carbon nanomaterials for use as MFC anodes. Chen et al. further extended the utilization of biomass sources, obtaining macroporous materials through high-temperature carbonization of sponge-like natural pomelo peel (Chen, 2012a), corrugated cardboard packing materials (Chen, 2012b), and crop plant kenaf (Chen, 2012c) as anode modification materials. The resulting macroporous substrates offered ideal environments for active bacterial growth, significantly augmenting bacterial attachment and EET efficiency, thereby leading to heightened power density outputs. Senthilkumar et al. (Senthilkumar et al., 2019) contributed to this trend by fabricating porous biochar (BC) materials through neem wood carbonization, subsequently modifying the biochar with cubic spinel-structured NiFe_2_O_4_ nanorods and the amorphous conducting polymer poly(3,4-ethylenedioxythiophene) (PEDOT), resulting in PEDOT/NiFe_2_O_4_/BC nanocomposites. This composite material exhibited good EET efficiency and enhanced ion diffusion due to the porous architectures of BC backbones and the tunnel structures within PEDOT/NiFe_2_O4/BC composites. The application of these nanocomposites as MFC anodes demonstrated a substantial improvement in MFC performance. Wang et al. (Wang et al., 2019) extended this approach, producing N,P-codoped macroporous carbonaceous materials through the one-step carbonization of pinecones, which facilitated the adhesion of electrochemically active microorganisms, accelerated MFC startup, and promoted EET efficiency. As a result, MFCs employing these anodes exhibited heightened voltage and power density outputs compared to those with conventional carbon felts.

Cathode materials play a critical role in microbial fuel cells (MFCs), particularly in the catalysis of the oxygen reduction reaction (ORR). Platinum has historically been the go-to catalyst for the ORR in MFCs due to its exceptional efficiency. While platinum (Pt)-based materials remain the benchmark for ORR catalysis, their drawbacks—such as limited availability, elevated costs, poor durability, and susceptibility to poisoning-induced inactivation—have prompted a fervent exploration of more sustainable alternatives (Wu et al., 2019; Xu et al., 2019; Zhang et al., 2021b). In this context, researchers are increasingly focusing on the development of advanced, cost-effective materials to supplant Pt-based counterparts. One such material exhibiting significant promise is carbon nanotubes (CNTs), which possess distinctive electrical and mechanical properties. These attributes position CNTs as candidates capable of facilitating electron transfer reactions when deployed as electrode materials in electrochemical processes. Through these explorations, the quest for cathode materials that balance catalytic performance, affordability, and sustainability remains a paramount priority in MFC research.

This manuscript presents significant advancements in the performance of air cathode membrane-less MFCs through the development of three-dimensional (3D) anodes derived from biomasses—specifically, corncobs and mango seeds. Additionally, an effective cathode alternative is introduced, utilizing an activated carbon-carbon nanotube composite to circumvent the need for expensive platinum-based cathodes. Remarkably, the newly developed electrode configurations were evaluated within sewage wastewater-driven MFCs without the introduction of external microorganisms or prior treatment of the wastewater. The proposed anodes were created by subjecting corncobs and mango seeds to controlled graphitization processes at elevated temperatures under inert atmospheres. Simultaneously, the advanced cathode was fabricated by depositing an ink blend comprising activated carbon, multiwall carbon nanotubes, polyvinylidene fluoride (PVDF), and dimethylformamide onto a carbon cloth substrate, followed by thermal treatment. The results are very promising as there was a high improvement in the generated power densities as well as a distinct decrease in the COD content.

## 2 Materials and methods

### 2.1 Anolyte solution

In El-Minya governorate, Egypt, a substantial water drainage system named MASRAF Al-MOHEET (GPS: 28.080493370742502, 30.717535901925135) extends over 135 km, receiving an influx of approximately 9,000 cubic meters per day comprising industrial, municipal, and agricultural wastewaters. Regrettably, these untreated pollutants are directly discharged into the Nile River, posing grave environmental hazards. To address this issue, the present study established a microbial fuel cell designed to operate with this specific wastewater. Samples were collected from the drainage system and subjected to comprehensive characterization using advanced instruments at the laboratories of the Sanitation and Drinking Water Company in El-Minya, Egypt. Details about the used instruments can be found in the Supporting Material file. The nature of this collected wastewater was deemed relatively aerobically treated due to its extraction from a flowing-water drainage channel. The summarized outcomes of these analyses are presented in Table 1.

**TABLE 1:** Characterization of the used sewage wastewater.

pH	COD (mg/L)	BOD (mg/L)	TSS (mg/L)	TDS (mg/L)	Total P (mg/L)	VSS (mg/L)	Total N (mg/L)	Alk (mg/L)
7.45 ± 0.03	305 ± 15	269 ± 5	65 ± 5	554 ± 17	3.594 ± 0.01	155 ± 3	4.3 ± 0.15	235 ± 7

COD, Chemical Oxygen Demand; BOD, biological oxygen demand; TSS, Total Suspended Solids; TDS, Total Dissolved Solid; Total P, Total phosphorus content; VSS, volatile suspended solids; Total N, Total nitrogen content; Alk, Total alkalinity.

The total count of bacteria was estimated using the following procedure: The as-collected sewage water was filtered to remove the solid particles. Later, 1 mL of the filtered water was added to 9 mL of sterilized water, shaken for 5 min, then the solution was diluted (10–1 to 10–6) and then the resulting solutions were plated directly onto the surface of nutrient agar (Vieira and Nahas, 2005). Incubate at 25 or 30°C for 10 days then, the colony forming units were counted (CFU). Data analysis was done using the SAS statistical programme (SAS Institute, Cary, NC, United States). Tukey’s estimates of honest significant differences (HSD) were computed from the ANOVA analysis when a significant F value was found. A level of statistical significance of 0.05 and 0.01 was established. The formula used to compute counts was *y* = log(*x*+1), where *x* was the initial CFU/mL sample of sewage water. The results indicated that the number of bacteria in used sewage was 10^5^∼10^6^ CFU/mL.

### 2.2 Anodes preparation

Harvested from rural areas, corncobs were meticulously polished and thoroughly cleansed with purified water. Subsequent to overnight drying at 80°C, the carbonization procedure was executed within a tube furnace, employing a gradual heating rate of 2° per minute and sustaining temperatures of 900°C, 1,000°C, and 1,100°C for a duration of 3 h. Prior to initiation, the tube was purged of air, and during the carbonization phase, the expulsion of gases was directed through a rubber conduit into a water basin to maintain atmospheric pressure within the tube. Visual documentation of the corncob both before and after the calcination process is provided in Supplementary Figure S1A of the Supporting Material. For the purpose of serving as a current collector, a stainless steel rod was integrated into the corncob structure (as depicted in the image). Subsequently, the prepared anode was positioned within an 80 mL anode chamber, as depicted in Supplementary Figure S1B.

Mango seeds were lightly polished, and then meticulously cleaned with clean water to get rid of any contaminants. After drying at 80°C overnight, the carbonization process was carried out in a tube furnace at a heating rate of 2 deg/min and holding for 3 h at different calcination temperatures; including 800, 900, 1,000, and 1,100°C. Images of the used mango seed taken before and after the calcination procedure are shown in Supplementary Figure S2 in the Supporting Material file. A stainless steel packed was inserted to act as a current collector.

### 2.3 Cathode preparation

An ink was prepared by dissolving 1 g from polyvinylidene fluoride (PVDF, Alfa Aesar, Germany) in 8 mL N,N dimethyl formmamide, then 0.5 g of activated carbon (AC) has been added. Influence of the carbon nanotubes content (CNTs, prepared in Jeonbuk University labs, Jeonju, South Korea) was investigated by preparing several inks containing 0, 0.01, 0.02, 0.035 and 0.05 g which represents 0, 2, 4, 7 and 10 wt% with respect to AC content, respectively. One face of carbon cloth sheet (CC, Electro Chem. Inc., United States) was well coated by the prepared ink and then subjected to drying at 60 C for 2 h. Later on, the cathodes were thermally treated at different temperatures; 25°C, 200°C and 350°C.

### 2.4 MFC construction

**FIGURE 1:**
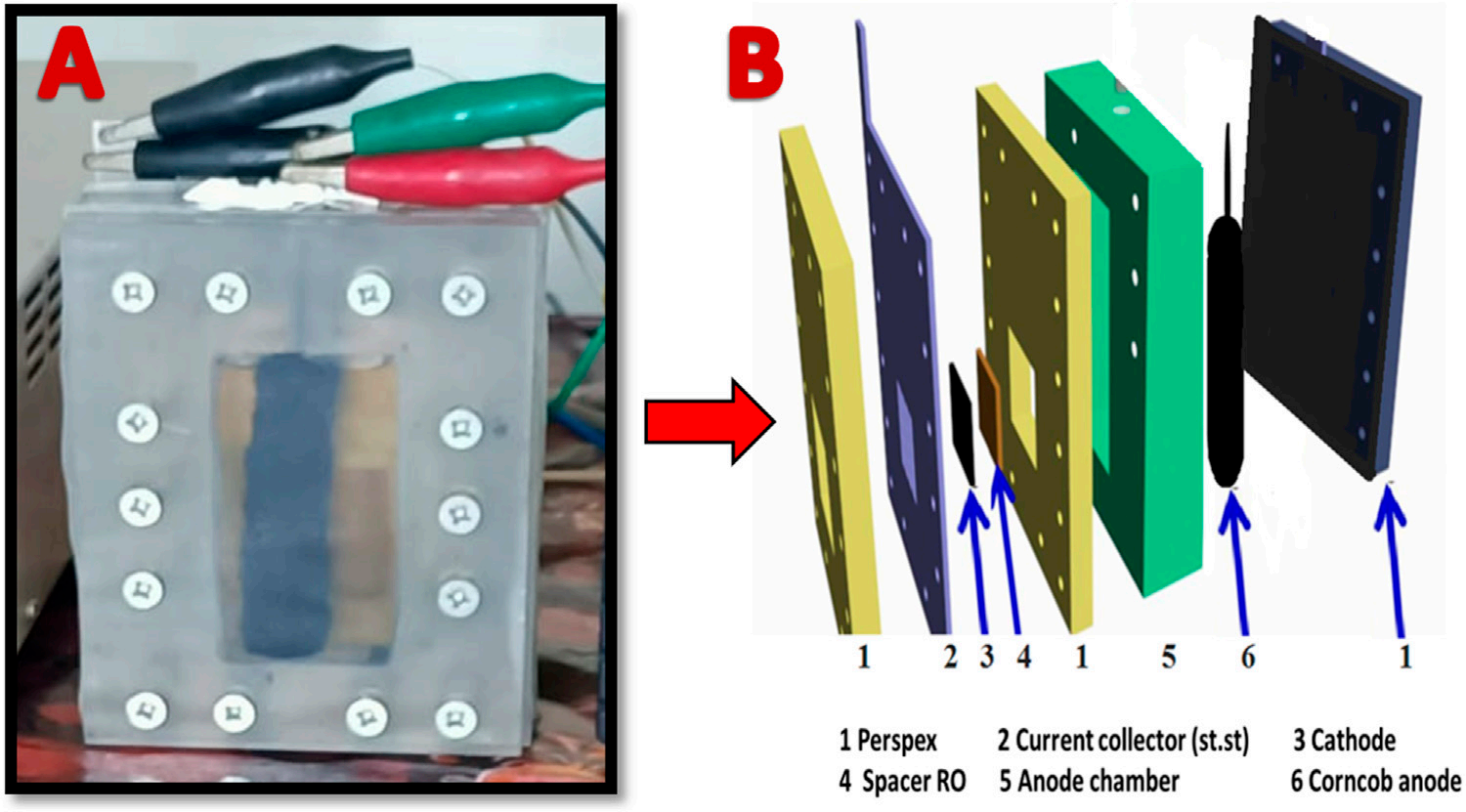
A photo image for the anode chamber in case of utilizing corncob-based anode; **(A)**, and conceptual illustration for the used MFC components; **(B)**.

To facilitate comparison, carbon cloth (CC) and carbon paper (CP) were procured from Electro Chem. Inc., in the United States and employed as anode materials. Moreover, to compare the performance of the proposed cathode, platinum-loaded carbon cloth (0.5 mg/cm^2^, Electro Chem Inc., United States) was invoked. Air-cathode, membrane-less single chamber style MFC was used in this study. The collected sewage wastewater has been filtered to remove the solid materials before using as anolyte. However, to enhance the electrical conductivity sodium acetate (5 g/L) was added to the wastewater (Barakat et al., 2023). Figure 1 displays the construction of used corncob anode-based MFC. As shown in Figure 1A, in case of corncob anode, a stainless steel (3 mm diameter) wire, inserted in the corncob (see Supplementary Figure S1 in the Supplementary Material), was serving as current collector. However, in case of the mango seed, the graphitized seeds were pressed in porous high corrosion resistance stainless steel basket which was exploited as a current collector; Supplementary Figure S2. The volume of anode chamber was about 80 mL, after filling, the solution was purged by nitrogen gas for 5 min to remove the dissolved oxygen.

Conducted at ambient room temperature, all experiments were carried out under anaerobic conditions. In the batch mode of the MFC (Supplementary Figure S3 in the Supporting Material file), the operational procedure involved running the cell to allow for the immobilization of microorganisms onto the anode. This process continued until the open circuit voltage (OCV) reached a stabilized state and equilibrium was achieved between the two half-reactions. The potential of the cell was monitored through a GL220 midi-logger, while the potentials at the anode and cathode were gauged using a reference electrode (Ag/AgCl). To assess the generated power density, linear sweep voltammetry (LSV) measurements were conducted with a scan rate of 1 mVs^−1^. In these electrochemical assessments, the working electrode was linked to the cathode, while the counter electrode and reference electrodes were connected to the MFC anode. A Potentiostat/Galvanostat (HA-1516, Japan) device was engaged to observe the current at 0 V, thus investigating the stability of the MFC under continuous mode conditions. This mode involved the cell functioning at a flow rate of 2 L/h via a peristaltic pump; Supplementary Figure S4 in the Supplementary Material. Both the current and power densities were adjusted in relation to the cathode’s surface area. In parallel, the measurement of chemical oxygen demand (COD) fluctuations entailed collecting MFC samples on a daily basis. Simultaneously, the assessment of current density occurred after the OCV attained stabilization within a continuous mode MFC.

### 2.5 Characterizations

The characterizations have been performed in the Central lab for Microanalysis and Nanotechnology, Minia University. The surface morphology of the fabricated anode material was meticulously examined through scanning electron microscopy (SEM) using a JEOL JSM-IT200 instrument (Japan). To gain insights into the chemical composition of the proposed anode, X-ray diffraction (XRD) analyses were conducted employing a Rigaku XRD instrument (Japan). Linear sweep voltammetry (LSV) measurements were performed to elucidate the electrochemical behavior of the anode material. This technique was executed using a VersaStat4 Potentiostat from AMETEK Scientific Instruments (United States), and a two-electrode configuration was adopted. In this configuration, the anode concurrently served as both the counter and reference electrode, while the cathode was designated as the working electrode. LSV was carried out at a scan rate of 1 mV/s to generate polarization curves, offering valuable insights into the electrochemical performance of the anode material. It is worth mentioning that the power and current densities were calculated based on the cathode area which is acceptable based on literature (Logan, 2008). Chemical oxygen demand (COD) measurements were conducted using DR38000 spectrophotometer using standard kites according to the standard procedure.

## 3 Results and discussion

### 3.1 Anodes characterizations

Graphitization of biomass involves subjecting the material to elevated temperatures under controlled conditions, leading to the breakdown of complex organic structures and the transformation into graphitic carbon. The process promotes the formation of a more ordered carbon structure, enhancing its electrical conductivity and promoting electron transfer kinetics in the microbial fuel cell (MFC) context. As the biomass undergoes graphitization, volatile components are driven off, leading to the development of voids or pores in the material (Yaqoob et al., 2021).

**FIGURE 2:**
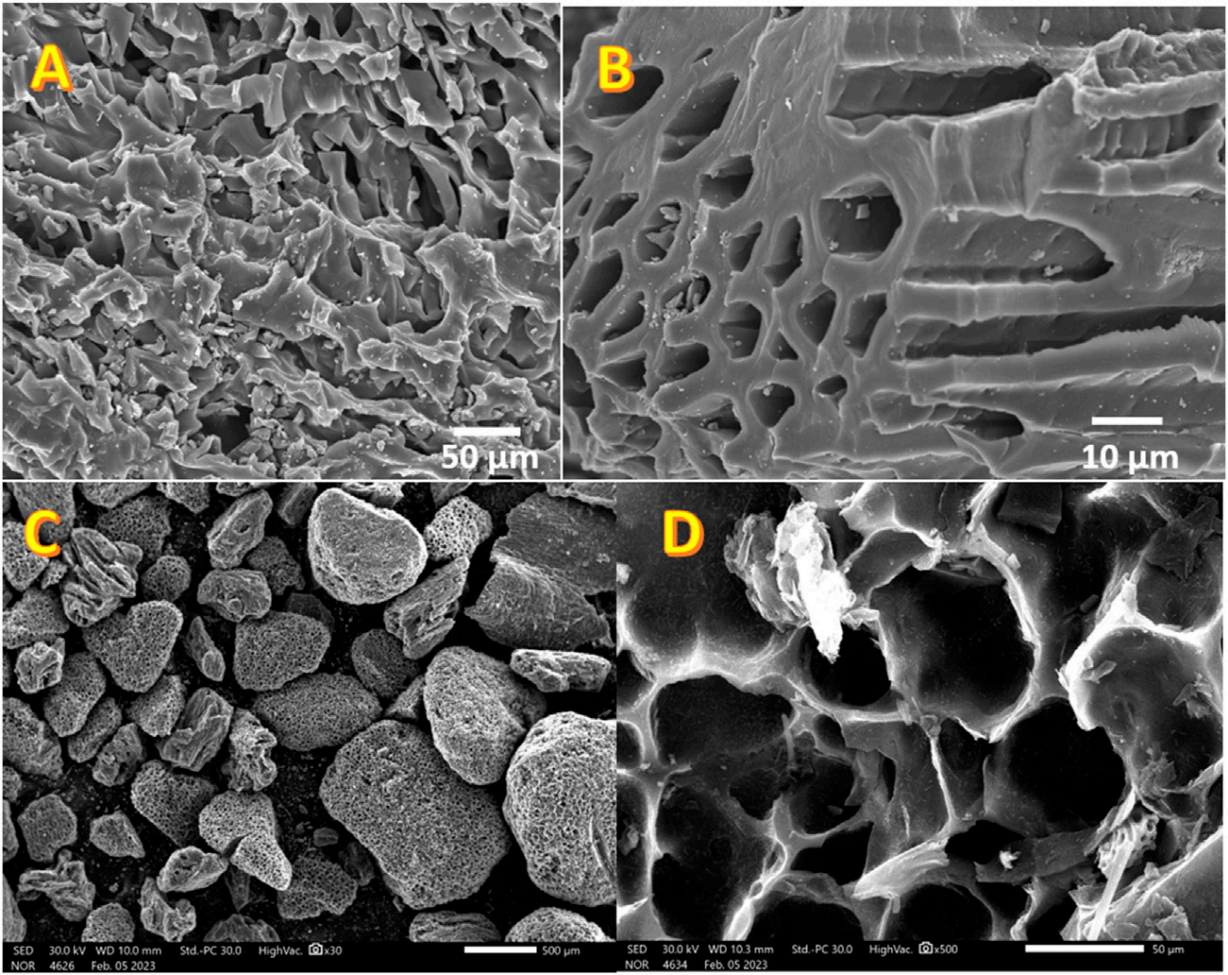
SEM and FE SEM images for the graphitized corncob; **(A, B)**, and mango seeds **(C, D)**.

The SEM and FESEM images presented in Figure 2 provide a comprehensive insight into the surface morphology of the graphitized corncob (Figures 2A, B) and mango seeds (Figures 2C, D). A noteworthy observation is that in the case of corncob, the pores exhibit greater depth and relatively smaller dimensions, whereas mango seeds exhibit larger, shallower pores. This discrepancy in pore characteristics can be attributed to the intrinsic structural differences between corncob and mango seeds, as well as the impact of the graphitization process. The formation of porous structures in graphitized biomasses is advantageous for their application as anodes in MFCs. These pores offer a multitude of benefits. Firstly, they increase the surface area available for microbial colonization, allowing for greater attachment and growth of electrochemically active microorganisms. This, in turn, enhances the catalytic activity of the anode by providing more sites for extracellular electron transfer. Secondly, the porous structure facilitates the diffusion of substrates and ions, enhancing mass transport within the anode material. This feature is vital for maintaining efficient microbial metabolism and electrochemical reactions. Moreover, the interconnected pore network aids in efficient gas exchange, ensuring that oxygen can effectively reach the microbial biofilm for the reduction reaction. In essence, the porous surface resulting from graphitization optimally combines high surface area, enhanced mass transport, and improved accessibility for microorganisms, collectively contributing to heightened MFC performance and power generation efficiency.

**FIGURE 3:**
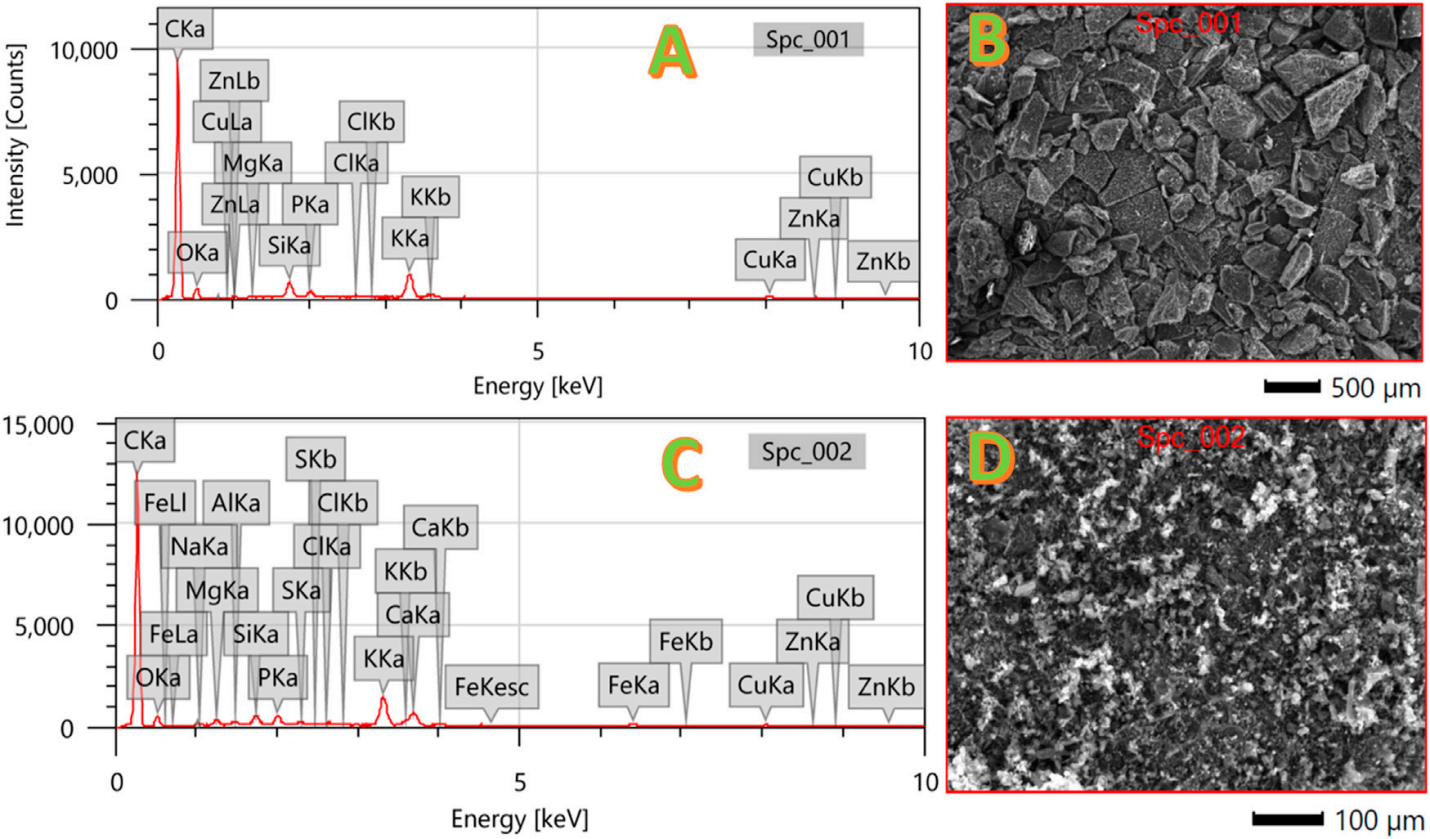
EDX analyses for the graphitized corncob; **(A, B)**, and mango seeds **(C, D)**.

The EDX analysis results, as depicted in Figure 3, provide essential insights into the elemental composition of both the corncob and mango seed anodes. The acquired spectra for both anodes unequivocally indicate the predominant presence of oxygen and carbon as the main constituents in each composition. This observation aligns well with the expected composition of carbonaceous materials and is in line with their role as anode materials in MFCs. Detailed elemental information is presented in Table 2, outlining the elements detected in the proposed anodes.

**TABLE 2:** Elemental composition for the proposed anodes (Wt.%).

	C	O	Na	Mg	Al	Si	P	S	Cl	K	Ca	Fe	Cu	Zn
Corncob	80.6	13.2	0.22	0.42	0.15	0.48	0.6	0.16	0.11	2.08	0.95	0.28	0.41	0.3
Mango seeds	81.2	14.5	—	0.13	—	0.85	0.44	—	0.12	1.97	—	—	0.48	0.42

Notably, the EDX analysis uncovers that potassium (K) is the most abundant metal detected in both the corncob and mango seed anodes, with percentages of 2.08 and 1.97 wt%, respectively. This relatively elevated potassium content can be attributed to the inherent composition of these natural biomasses, as potassium is a common element found in plant-based materials. Moreover, these results reinforce the viability of utilizing these biomass-derived materials as anode candidates within MFCs, given their natural potassium content that can contribute to catalytic activity enhancement and microbial growth stimulation.

A noteworthy distinction arises from the comparison between the two anodes. The analysis discloses that, in addition to the elements detected in the mango seed anode, the corncob anode harbors additional elements including sodium (Na), sulfur (S), aluminum (Al), and iron (Fe). This dissimilarity may stem from variations in the growth environment and elemental composition of the raw materials themselves. The presence of these elements underscores the complex nature of biomass-derived materials and their inherent heterogeneity. While the detected elements within the corncob anode are trace constituents, they might impart subtle effects on its electrochemical properties and overall performance in MFCs.

**FIGURE 4:**
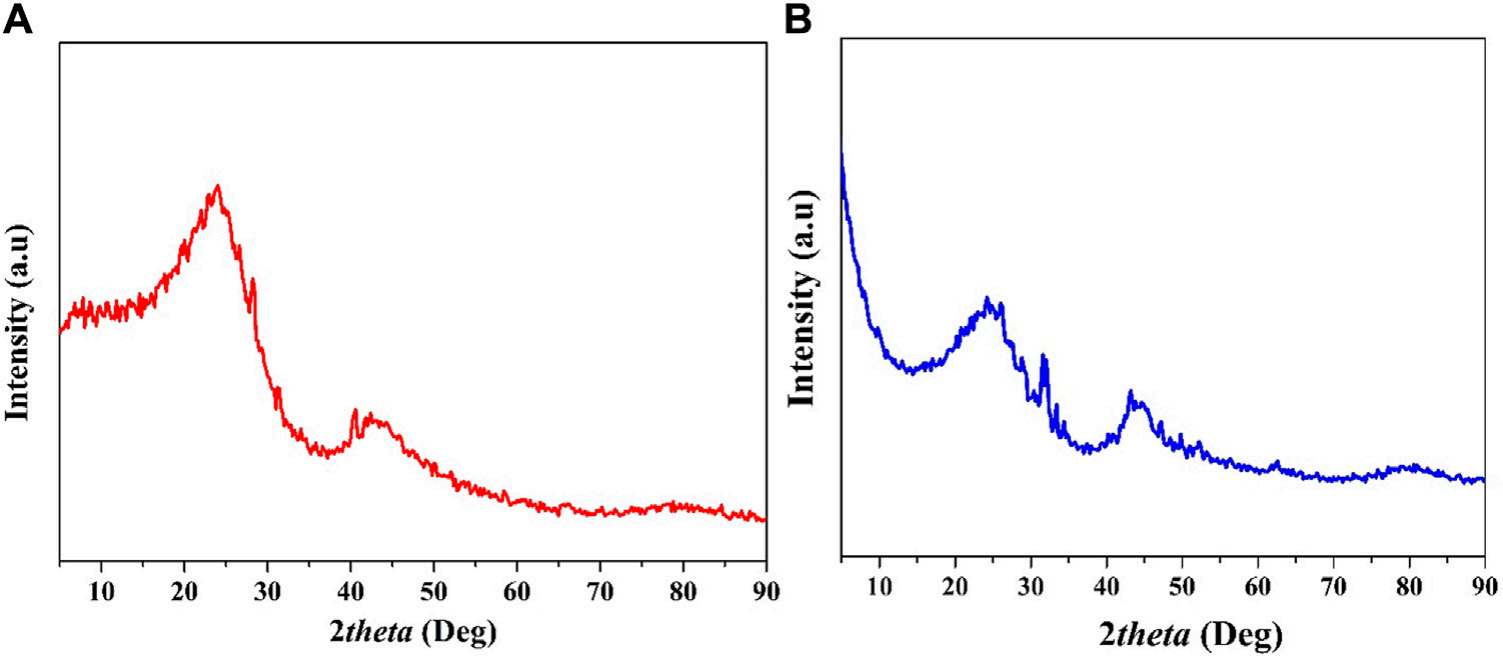
XRD patters for the graphitized corncob; **(A)**, and mango seeds **(B)**.

The XRD results, as depicted in Figure 4, provide insightful details concerning the crystalline structure of both the corncob and mango seed anodes, which were prepared through similar graphitization processes. The XRD patterns exhibit characteristic peaks centered around 24° and 43°, which correspond to the (002) and (100) lattice planes, respectively. This pattern closely resembles the findings observed for the mango seed anode prepared at 1,100°C. The presence of these characteristic peaks in both anode materials underscores the development of a pseudo-graphitic carbon structure resulting from the graphitization process. The emergence of a graphitic-like arrangement signifies the alignment of carbon atoms in a layered structure, akin to that found in conventional graphitic materials (Liu et al., 2012). This arrangement, characterized by a graphitic-like structure, contributes to heightened electrical conductivity within the anode, which in turn facilitates the efficient movement of electrons through the material. This enhanced electrical conductivity is particularly advantageous for MFC applications, as it promotes effective electron transfer from the microorganisms to the anode’s surface (Zhang et al., 2010).

The study’s findings emphasize the pivotal role of pyrolysis temperature in the graphitization process and its impact on anode properties. Notably, a higher pyrolysis temperature proves beneficial for achieving the pseudo-graphitization of both corncob and mango seed anodes. This process significantly enhances the anodes’ electrical conductivity, consequently amplifying their electron transfer capabilities. Improved conductivity enables the more efficient utilization of electrons generated during microbial oxidation within MFCs. Consequently, the selection of an optimal pyrolysis temperature emerges as a crucial factor in tailoring the electrical properties of anode materials for optimal MFC performance. These findings underscore the importance of the graphitization process and underscore the significant role that elevated pyrolysis temperatures play in augmenting the conductivity and overall effectiveness of both corncob and mango seed anodes in the context of MFC applications (Huang et al., 2013; Lai et al., 2016; Subramani et al., 2017; Wickramaarachchi et al., 2021).

**FIGURE 5:**
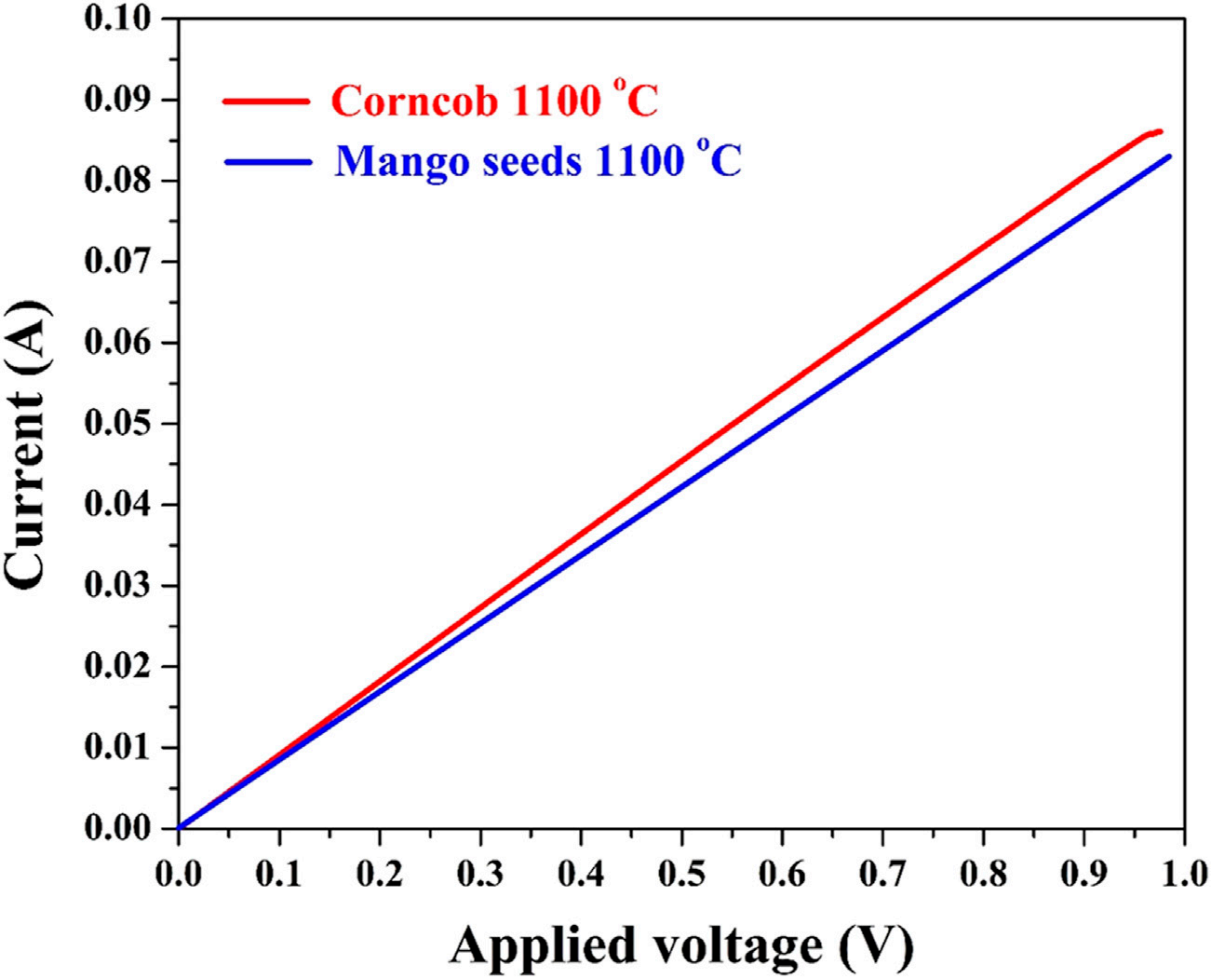
I–V relationship for the carbonized corncob and mango seeds at 1,100°C. The measurement was conducted with a 1-cm two electrode setup at seven different locations at the same anode; the plotted data are the average values.

The significance of electrical conductivity as a fundamental attribute of anode materials cannot be understated. The I–V plot presented in Figure 5, depicting average values derived from seven measurements for each anode, provides critical insights into the electrical behavior of the corncob and mango seed anodes. Notably, the displayed data reveal that both anodes exhibit conductivity akin to that of metals, a characteristic that holds substantial implications for their performance within microbial fuel cells (MFCs).

The determined low electrical resistance, approximately 1.2 ± 0.01 Ω, underscores the excellent electrical conductivity of both anode materials. This exceptional conductivity ensures efficient electron transport throughout the anode matrix, a crucial aspect for sustaining and optimizing electrochemical reactions within MFCs. The observed metal-like conductivity in the anodes further underscores their potential to serve as efficient electron conduits. In MFCs, where bioelectrochemical reactions are reliant on the seamless flow of electrons, materials with robust electrical conductivity are essential for minimizing energy losses and maximizing power generation. The low electrical resistance values demonstrated by the corncob and mango seed anodes signify that these materials offer exceptional pathways for electron movement, fostering efficient charge transfer between the microbial biofilm and the anode surface.

### 3.2 Carbon nanotubes/activated carbon cathode characterization

**FIGURE 6:**
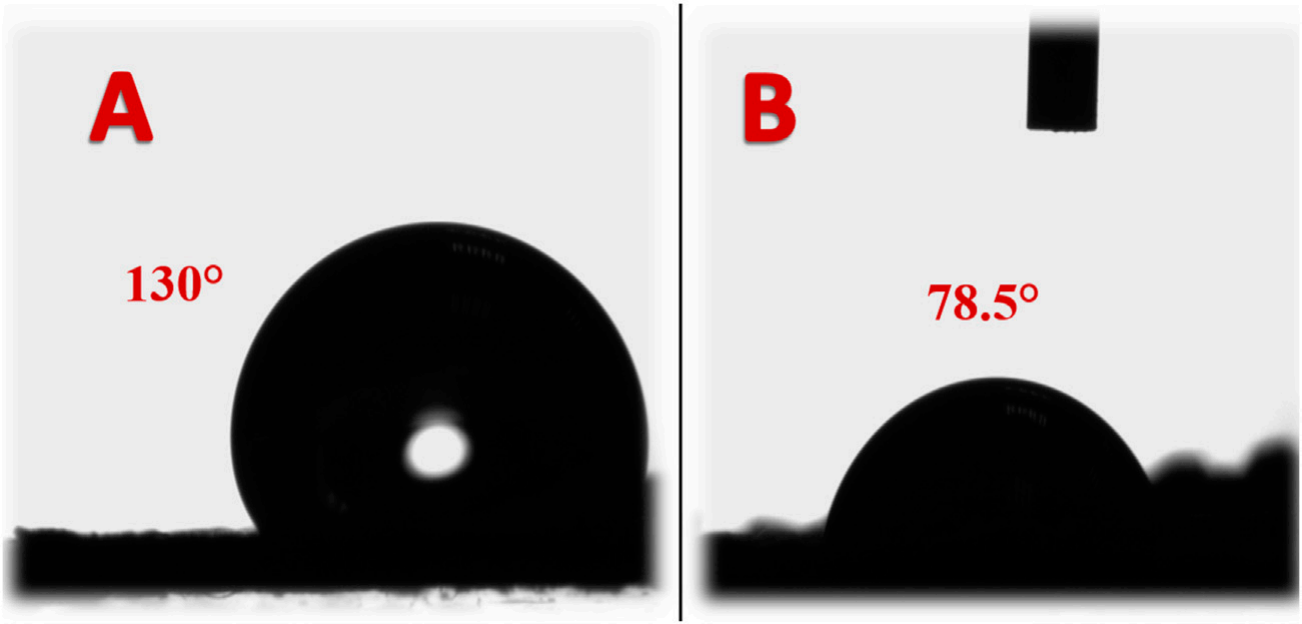
Water contact angle for pristine AC; **(A)** and AC/CNTs (7%); **(B)**.

The measurement of water contact angle serves as a pivotal parameter, particularly for cathodes in MFCs, where interfacial interactions play a crucial role in facilitating electrochemical reactions. The results, illustrated in Figure 6, present a compelling insight into the impact of incorporating carbon nanotubes (CNTs) on the water contact angle of cathodes. Notably, the comparison between CNTs-incorporated and CNTs-free cathodes, both prepared through a similar procedure and thermally treated at 350°C, reveals a significant transformation in surface characteristics. This transformation is highlighted by the alteration from hydrophobic to hydrophilic behavior upon the inclusion of CNTs. Quantitatively, the measured water contact angles of 130° and 78.5° for pristine and CNTs-incorporated cathodes, respectively, affirm the hydrophilic transformation induced by CNTs. The decrease in water contact angle upon CNTs incorporation indicates an enhanced wettability of the cathode surface. This shift towards hydrophilicity holds notable implications for MFC performance.

In microbial fuel cells, where cathodes facilitate the reduction of oxygen, a hydrophilic surface offers several advantages. First, a hydrophilic cathode surface promotes improved mass transport of oxygen, enabling a higher availability of the reactant at the cathode-electrolyte interface (Li et al., 2016). This, in turn, enhances the rate of oxygen reduction reactions and overall cathodic performance. Second, a hydrophilic surface mitigates the accumulation of liquid water and gas bubbles on the cathode surface. The reduction in gas bubble formation prevents potential blockages that could hinder oxygen diffusion, ensuring consistent and unhindered oxygen supply to the cathode (Pang et al., 2018).

The pivotal function of the cathode in fuel cells, including microbial fuel cells (MFCs), lies in catalyzing the oxygen reduction reaction (ORR), a fundamental step that determines overall cell performance. The mechanism of the ORR is significantly influenced by the pH of the medium, making it crucial to assess the performance of proposed cathode materials under relevant conditions. In the case of the investigated MFC utilizing sewage water, this solution was employed to evaluate the catalyst’s performance in both *in-situ* and *in-vivo* scenarios, representing outside and inside the MFC, respectively.

**FIGURE 7:**
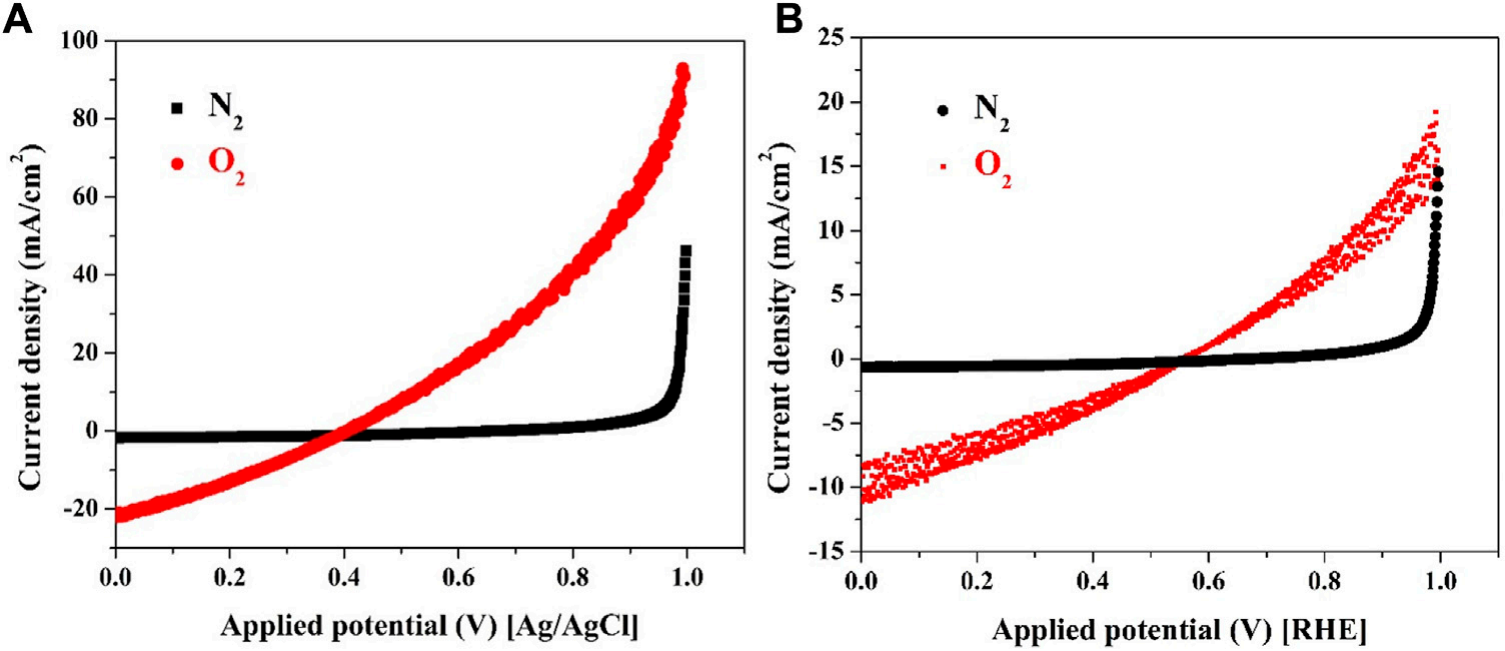
Linear sweep voltammetry in presence of O_2_ and N_2_ bubbling for CNTs/AC mixture (7 wt% sample) when the mixture was deposited on the surface of glassy carbon electrode; **(A)**, and on carbon cloth (real cathode thermally treated at 350°C); **(B)**.

Two distinct experimental setups were employed for investigating the catalyst’s performance in the *in-situ* mode. Firstly, a three-electrode cell was used, where a glassy carbon (GC) electrode acted as the working electrode, with Ag/AgCl and Pt electrodes serving as reference and counter electrodes, respectively. A working electrode was fabricated by depositing a layer of CNTs/activated carbon (AC) mixture (7 wt% CNTs) onto the GC surface. Typically, 0.002 g from CNTs/AC mixture (7 wt% CNTs) was dispersed in 20 µL Nafion solution (5% in isopropanol) and 400 µL isopropanol. Then 15 µL from this suspension has been poured on the active area of the GC which was subjected to drying process at 80°C before use, the linear sweep voltammetry (LSV) results in presence of nitrogen and oxygen bubbling are depicted in Figure 7A. In the second experiment, the prepared cathode was integrated as the working electrode in a two-electrode cell configuration, where the counter and reference electrodes were connected to a stainless steel bar, Figure 7B.

The obtained results, depicted in Figure 7, reflect the electrode’s performance under various conditions. Notably, during nitrogen bubbling, no current was generated in both the three-electrode and two-electrode setups. However, when the solution became saturated with oxygen through bubbling, an observable current was recorded in both experiments. These findings underscore the electroactivity of the used cathode material for the oxygen reduction reaction. The detected current in the presence of oxygen demonstrates the catalyst’s effective catalytic activity, showcasing its ability to facilitate the ORR. Moreover, the observed onset potential, representing the cathode potential where the ORR initiates, was notably favorable at 0.39 and 0.544 V vs. Ag/AgCl and reversible hydrogen electrode (RHE) reference electrodes, respectively. This indicates that the proposed cathode material exhibits a high catalytic efficiency and an advantageous electrocatalytic behavior towards oxygen reduction. The results highlight the potential of the developed cathode material as an efficient catalyst for the ORR in MFCs. The observed onset potentials and current generation under oxygen-saturated conditions further emphasize its electrocatalytic prowess, rendering it a promising candidate for enhancing the oxygen reduction process within microbial fuel cells.

### 3.3 Proposed anodes performance in standard Pt/C cathode-based MFCs

#### 3.3.1 Effect of calcination temperature

**FIGURE 8:**
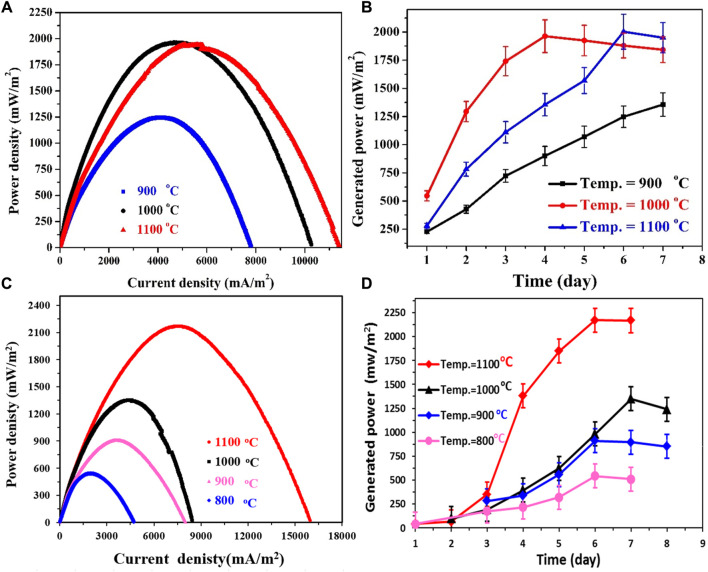
Effect of calcination temperature on the generated power density from sewage wastewater-driven microbial fuel cells using corncob; **(A)** and mango seeds; **(C)**, Pt/C-loaded carbon cloth was used as cathode. **(B, D)** display the generated power densities for several days from corncob and mango seed–based MFCs, respectively.

The investigation into graphitization temperature assumes significance due to its impact on the physicochemical attributes of the proposed anodes. To comprehensively assess this influential parameter, the corncob-based anodes were fabricated at three distinct temperatures: 900, 1,000, and 1,100°C. These prepared anodes were subsequently utilized to assemble three separate microbial fuel cells using standard Pt/C as a cathode. The performance outcomes of these cells are vividly presented in Figures 8A, B. Notably, the polarization curves depicted in Figure 8A reveal commendable performance across all three anodes. However, the findings suggest a preference for calcination temperatures surpassing 1,000°C. Specifically, the corncob anodes graphitized at 1,000°C and 1,100°C exhibit almost identical performance, as evidenced by the closely matched power densities generated by the respective cells. In quantifiable terms, the maximum power density attained from the MFCs employing corncob anodes graphitized at 1,000°C and 1,100°C registers at 1905 ± 90 and 2003 ± 125 mW/m^2^, respectively. Nonetheless, a drop in the calcination temperature to 900°C results in a diminished maximum generated power density of 1,250 ± 85 mW/m^2^. This disparity in performance can be ascribed to variations in the electrical conductivities of the fabricated anodes, as previously elucidated.

Monitoring the power density evolution over an 8-day period (Figure 8B) unveils a correlation between stabilization time and graphitization temperature. Notably, the anodes prepared at higher calcination temperatures achieve their maximum power densities within the course of the investigation; specifically, at 4 and 6 days for anodes graphitized at 1,000°C and 1,100°C, respectively. In contrast, the lower temperature-anode demonstrates a progressive increment in power density throughout the observation period. This phenomenon provides an additional perspective on the relatively modest performance of the 900°C-anode. The lingering presence of functional groups on the anode surface could potentially hinder microorganism attachment, contributing to the observed disparities in performance.

To explore the impact of graphitization temperature on mango seed anodes, the seeds were subjected to preparation at four distinct temperatures: 800, 900, 1,000, and 1,100°C. The outcomes of these variations are depicted in Figure 8C, offering valuable insights into their performance. An examination of the polarization curves in Figure 8C reveals that the anodes produced at 1,100°C and 1,000°C exhibit favorable performance, while those prepared at 900°C and 800°C demonstrate relatively lower efficacy. Specifically, the power densities generated by the 1,100°C and 1,000°C anodes amount to 2,170.8 ± 90 and 1,350.6 ± 125 mW/m^2^, respectively. However, a reduction in the calcination temperature to 900°C and 800°C results in a decline of the maximum power density to 895.4 and 542.6 mW/m^2^, respectively.

Notably, a noteworthy distinction arises in the time required for the anodes to attain their maximum power density, as illustrated in Figure 8D. The 1,000°C anode necessitated 7 days to achieve peak performance, whereas the MFCs employing the 1,100, 900, and 800°C anodes reached their highest power densities within 6 days. This temporal variation offers intriguing insights into the performance and stability of distinct anode materials within microbial fuel cells (MFCs).

The attainment of maximum power density duration can be influenced by multiple factors, encompassing initial biofilm formation, the establishment of efficient electron transfer networks, and the acclimatization of microorganisms to the anode surface. The lag in reaching peak power density for the 1,000°C anode suggests a prolonged period required for biofilm development, electron transfer network establishment, and microbial adaptation to the anode surface. This delay in initial performance could be attributed to the specific surface properties or structural attributes of the 1,000°C anode, potentially influencing microorganism attachment and growth.

In contrast, MFCs employing the 1,100°C, 900°C, and 800°C anodes achieved their maximum power densities within 6 days. This expedited timeframe implies that these anode materials fostered more rapid biofilm establishment, electron transfer network formation, and microbial adaptation. The surface features observed in SEM images, such as layered branches and micro-sized deep holes, likely played a pivotal role in providing favorable sites for microorganism attachment, thereby promoting biofilm development. This robust biofilm formation facilitated the creation of an effective conductive network, thereby enabling efficient electron transfer and enhanced power generation. These insights underscore the significance of optimizing anode materials and their surface characteristics to promote efficient biofilm development and electron transfer, ultimately enhancing power generation in MFCs.

**FIGURE 9:**
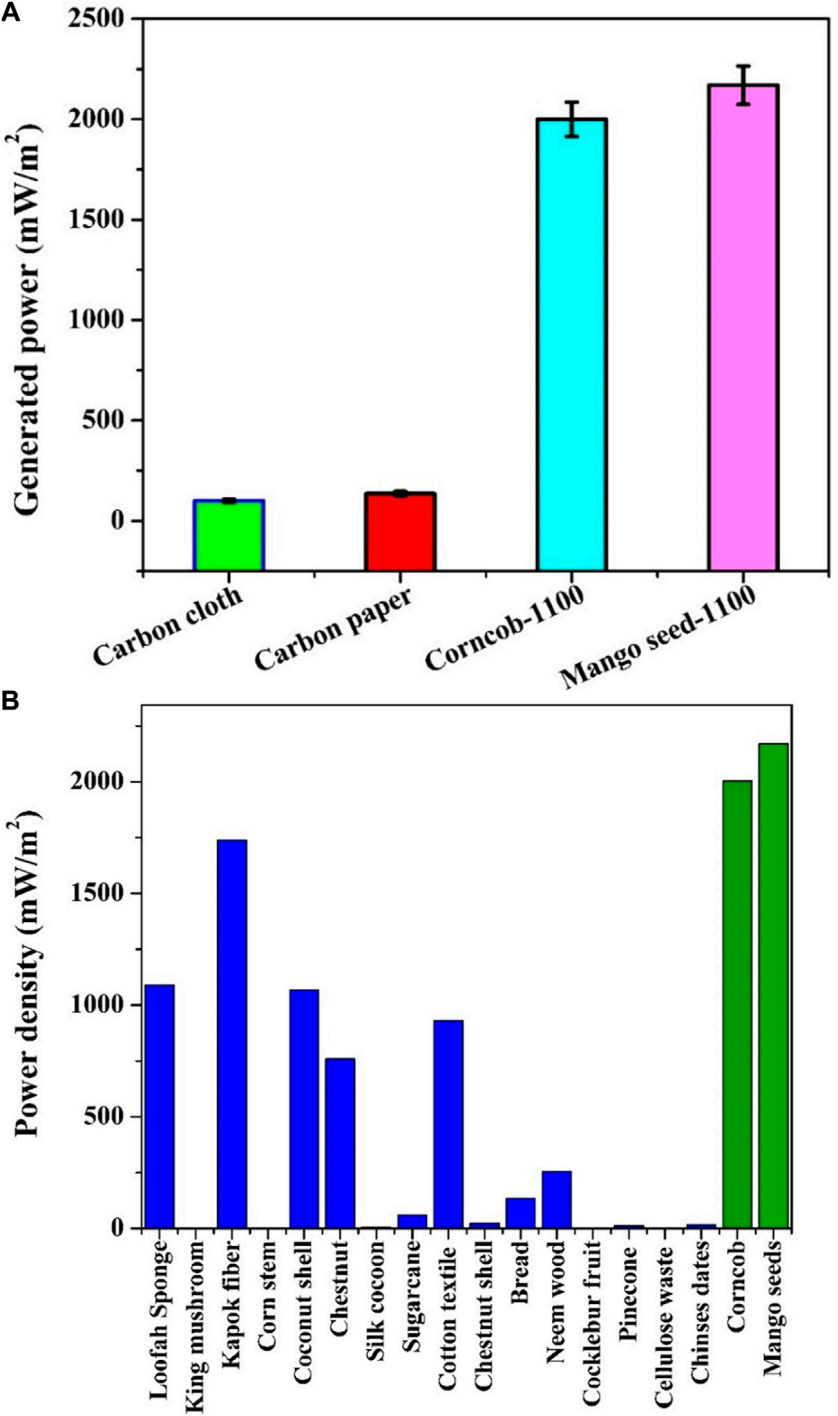
Comparison between the proposed graphitized corncob and mango seeds anodes and the commercial ones (carbon close and carbon paper) based on the generated power density from sewage wasetwater-driven microbial fuel cells using Pt/C standard cathodes; **(A)** and comparsion with recently reported anodes; **(B)** Refernces in sequence: Loofah; 2013 (Yuan et al., 2013), 2015 (Karthikeyan et al., 2015), 2014 (Zhu et al., 2014), 2015 (Karthikeyan et al., 2015), 2015 (Yuan et al., 2015), 2016 (Chen et al., 2016), 2016 (Lu et al., 2017), 2017 (Zhou et al., 2017), 2018 (Zeng et al., 2018), 2018 (Chen et al., 2018), 2018 (Zhang et al., 2018; Senthilkumar et al., 2019), 2019 (Yang et al., 2019), 2019 (Wang et al., 2019), 2020 (Yaqoob et al., 2020), Chinese dates; 2022 (Meng et al., 2022).

The comparison depicted in Figure 9A offers a striking insight into the performance disparities between the proposed anodes and commercial alternatives (carbon cloth and carbon paper) within air cathode, sewage water-driven MFCs. Remarkably, the utilization of the proposed anodes showcases a substantial escalation in the generated power density, distinctly surpassing that achieved by commercial counterparts. Notably, adopting the proposed anodes yields a remarkable twenty-fold increase in the generated power density, underscoring their superior performance in this context. To provide a quantitative perspective, the power density attained with carbon cloth, carbon paper, graphitized corncob-1100, and graphitized mango seeds-1100 were recorded as 100, 135, 1963, and 2,171 mW/m^2^, respectively.

Figure 9B extends the comparative analysis by juxtaposing the proposed anodes with recently developed biomass-driven anodes. It is important to note that these prior anodes were often deployed with simulated anolytes, frequently comprising acetate, and some featured specific electron acceptor compounds within the cathode chamber (e.g., Potassium ferricyanide). In light of these distinctions, it is evident that the proposed anodes possess a distinct advantage and hold a prominent position as highly recommended anode materials.

The superior performance of the proposed anodes can be attributed to their specific attributes and preparation processes. The graphitized nature of the anodes, achieved through controlled high-temperature carbonization, enhances their electrical conductivity and facilitates efficient electron transfer between microorganisms and the anode surface. Additionally, the porous structures observed in the SEM images likely provide favorable sites for microbial attachment, thereby promoting effective biofilm formation and contributing to improved power generation. Furthermore, the utilization of natural biomasses as precursors not only offers an eco-friendly and cost-effective approach but also introduces inherent functionalities that can positively influence microbial adhesion and electron transfer.

The distinct advantage of the proposed anodes in a real sewage water environment is a significant step forward in advancing the practical application of microbial fuel cells for wastewater treatment and energy generation. The demonstrated performance highlights the potential of these anodes to contribute to sustainable and efficient MFC systems, further bolstering their appeal for broader implementation in various environmental and energy-related contexts.

### 3.4 New cathode performance

#### 3.4.1 Effect of treatment temperature

**FIGURE 10:**
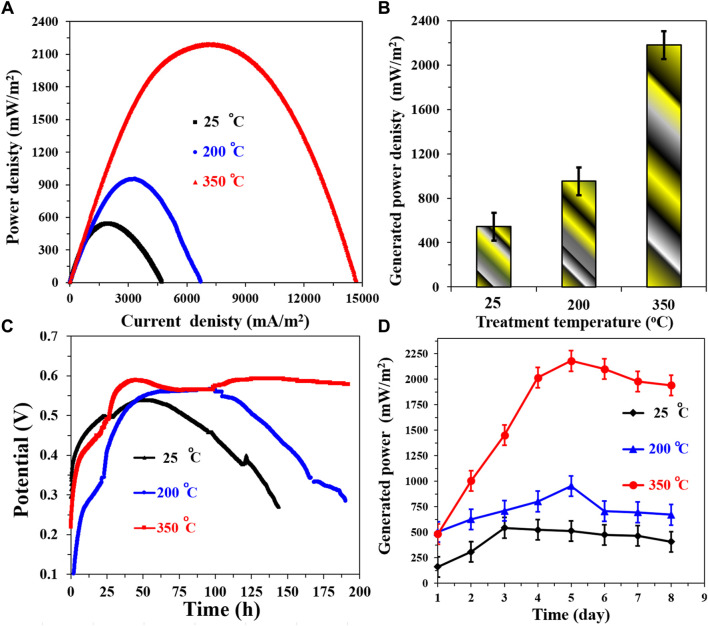
Influence of the treatment temperature on the performance of the proposed CNTs/AC cathode in a corncob-1000 anode. **(A)** Polarization curves of the assembled microbial fuel cells, **(B)** Maximum generated power densities at different cathode treatment temperatures, **(C)** The change of the open cell potential with time, and **(D)** Influence of running time on the generated powers from the assembled MFCs.

The investigation into the influence of heat treatment temperature on the newly proposed CNTs/AC cathode yielded valuable insights into its performance characteristics. By subjecting prepared cathodes containing 7 wt% CNTs to different treatment temperatures (25°C, 200°C, and 350°C), a comprehensive understanding of the thermal effects on cathode performance was obtained. Figure 10A illustrates the polarization curves for MFCs assembled using the treated cathodes paired with the corncob-1000°C anode. Notably, an intriguing trend emerges as the treatment temperature increases, the maximum power density also exhibits a corresponding increase.

The generated power density values depicted in Figure 10B quantitatively confirm this observation. The trend is clear: cathodes treated at 25°C, 200°C, and 350°C yield generated power densities of 542 ± 7, 953 ± 9, and 2,178 ± 15 mW/m^2^, respectively. This phenomenon can be attributed to multiple factors. The treatment temperature appears to facilitate a more optimized distribution of the CNTs/AC mixture and potentially promotes enhanced adhesion to the substrate. In the context of lithium-ion batteries using PVDF as a binder, heat treatment has been observed to improve the adhesion of active materials to electrodes and enhance cycling performance due to uniform binder distribution on active material particles (Li et al., 2008).

Intriguingly, the performance enhancement is also reflected in the stability of the cathodes. Figure 10C presents changes in the open circuit voltage (OCV) over time. The results clearly highlight the superior stability of cathodes treated at 350°C compared to those treated at lower temperatures. The decay of OCV is delayed and reduced as the treatment temperature increases. This suggests that higher treatment temperatures facilitate a more stable and resilient cathode structure, preventing degradation over extended operational periods (Marshall et al., 2021).

The dynamic behavior of the generated power densities with time, as illustrated in Figure 10D, further supports the performance trends. The MFC assembled with the untreated cathode exhibited the lowest power density, achieving only 542 ± 7 mW/m^2^. In contrast, both 200°C and 350°C treatment temperatures resulted in more robust power density profiles. The 350°C cathode-based MFC demonstrated a gradual increase until reaching a peak on the fifth day, followed by a decrease attributed to the depletion of organic compounds as the cell operated in batch mode. The 200°C cathode-based MFC exhibited a similar behavior, albeit with a slower increase rate and earlier peak. The robustness of power density profiles with the treated cathodes reinforces the notion that heat treatment enhances both stability and efficiency of the cathode. Overall, these results underscore the crucial role of heat treatment temperature in optimizing the performance of the newly proposed CNTs/AC cathode. This thermal treatment appears to induce structural changes that improve the distribution of active materials and binder, leading to enhanced adhesion, stability, and ultimately, increased power generation in the microbial fuel cells (Yang et al., 2014).

#### 3.4.2 Effect of carbon nanotubes content

**FIGURE 11:**
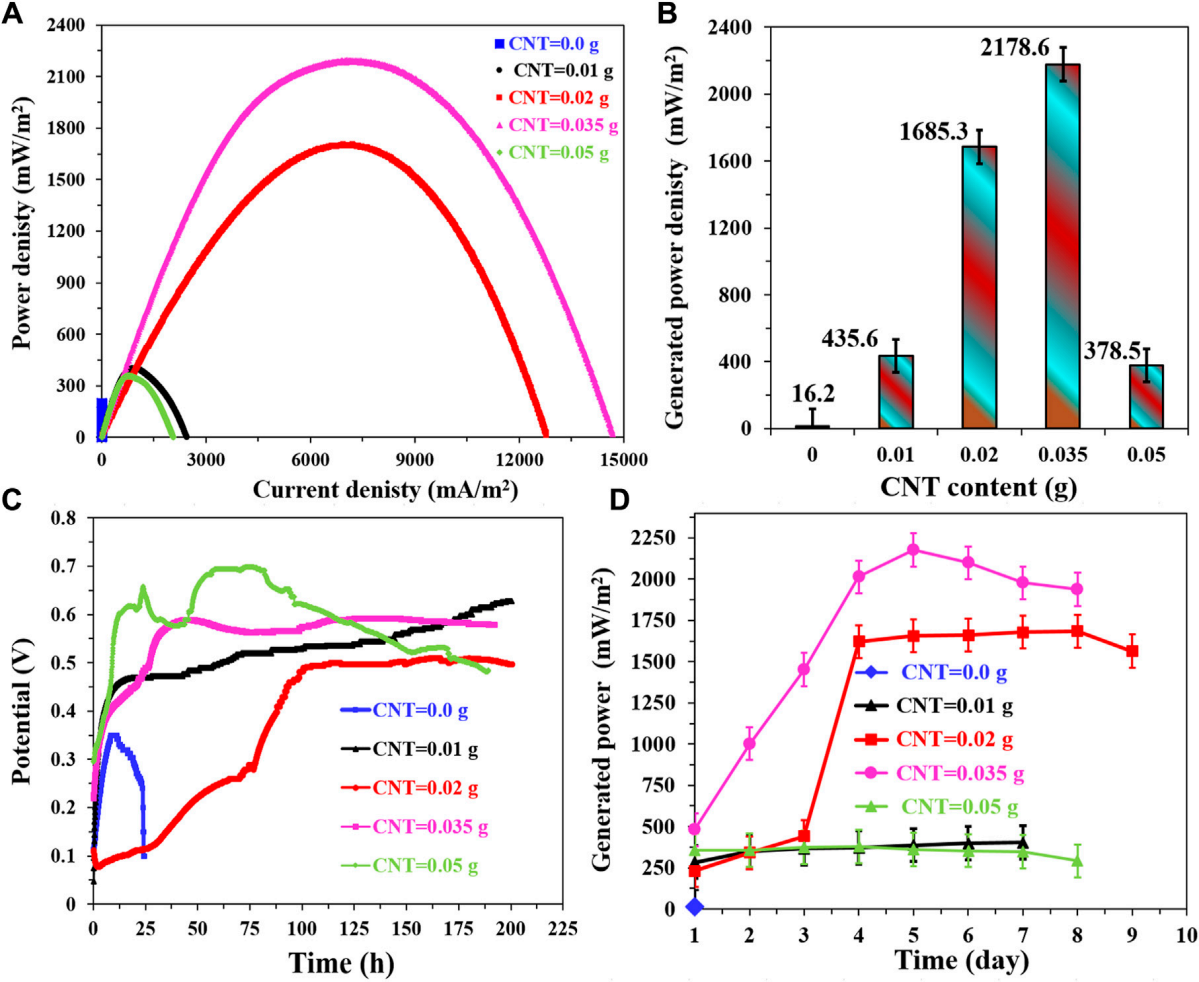
Influence of CNTs content on the performance of the proposed cathode (1 g polymer +0.5 g AC) treated at 350°C in corncob‒1000°C anode‒based microbial fuel cells, using NaAc (5 g/L)/sewage wastewater anolyte. **(A)** Polarization curves of the assembled microbial fuel cells, **(B)** Maximum generated power densities at different CNTs contents, **(C)** the change of the open cell potential with respect with the time and **(D)** influence of running time on the generated power densities from the assembled MFCs.

The utilization of carbon nanotubes (CNTs) as electrode materials offers distinct advantages owing to their unique electrical and mechanical properties, which facilitate efficient electron transfer reactions in various electrochemical processes (Patolsky et al., 2004; Deng et al., 2010). In this study, the potential of CNTs to catalyze the oxygen reduction reaction (ORR) was harnessed, designating CNTs as the primary catalyst and main cathode material. The investigation into the influence of CNTs content on the performance of prepared cathodes treated at 350°C, while employing the corncob-1000 anode, yielded informative insights, as presented in Figure 11.

Figure 11A showcases the polarization curves, illustrating the profound impact of CNTs content on cathode performance. The power density generated by the microbial fuel cells (MFCs) exhibited considerable variations, contingent on the CNTs content. This effect is quantified in Figure 11B, where the maximum power density of 2,178.6 ± 25 mW/m^2^ corresponds to a CNTs content of 0.035 g (7% with respect to AC). The observed minimal power density of 16 ± 0.7 mW/m^2^ from the MFC containing a CNTs-free cathode validates the electrocatalytic activity of CNTs in facilitating the ORR reaction. Furthermore, the augmentation of CNTs content up to the optimal value yields an enhancement in generated power, whereas a further increase (to 0.05 g, 10 wt%) leads to a diminishing performance, indicating a non-linear relationship between CNTs content and cathode efficiency.

Figure 11C provides insight into the stability of the assembled MFCs, showcasing the changes in open circuit voltage (OCV) over time. Notably, excluding 0 and 10 wt% CNTs content, the OCV remains stable. Conversely, the CNTs-free cathode experiences rapid OCV decay, underscoring the critical role of CNTs in sustaining cathode stability. Similarly, the MFC with 10 wt% CNTs content displays a gradual OCV decrease after reaching its maximum value of 7 V. Figure 11D further characterizes the generated power densities with time. The absence of CNTs leads to a cessation of generated power, while stable and high-power densities are achieved after the fourth day in the MFCs equipped with 4 and 7 wt% CNTs-containing cathodes, with the latter exhibiting superiority. In contrast, the MFCs with 2 and 10 wt% CNTs content cathodes maintain stable but notably lower power densities compared to their counterparts.

The observed trends can be attributed to the intricate interplay of factors such as CNTs’ electrocatalytic properties, their distribution within the cathode matrix, and the achievable balance between enhanced ORR kinetics and electron transfer efficiency. The optimal CNTs content of 7 wt% results in the highest power density, indicating an optimal configuration that ensures efficient oxygen reduction while avoiding the potential hindrance of excessive CNTs content. These findings underscore the importance of precisely tailoring CNTs content for optimizing cathode performance in microbial fuel cells.

#### 3.4.3 Comparison with standard Pt/C cathode

**FIGURE 12:**
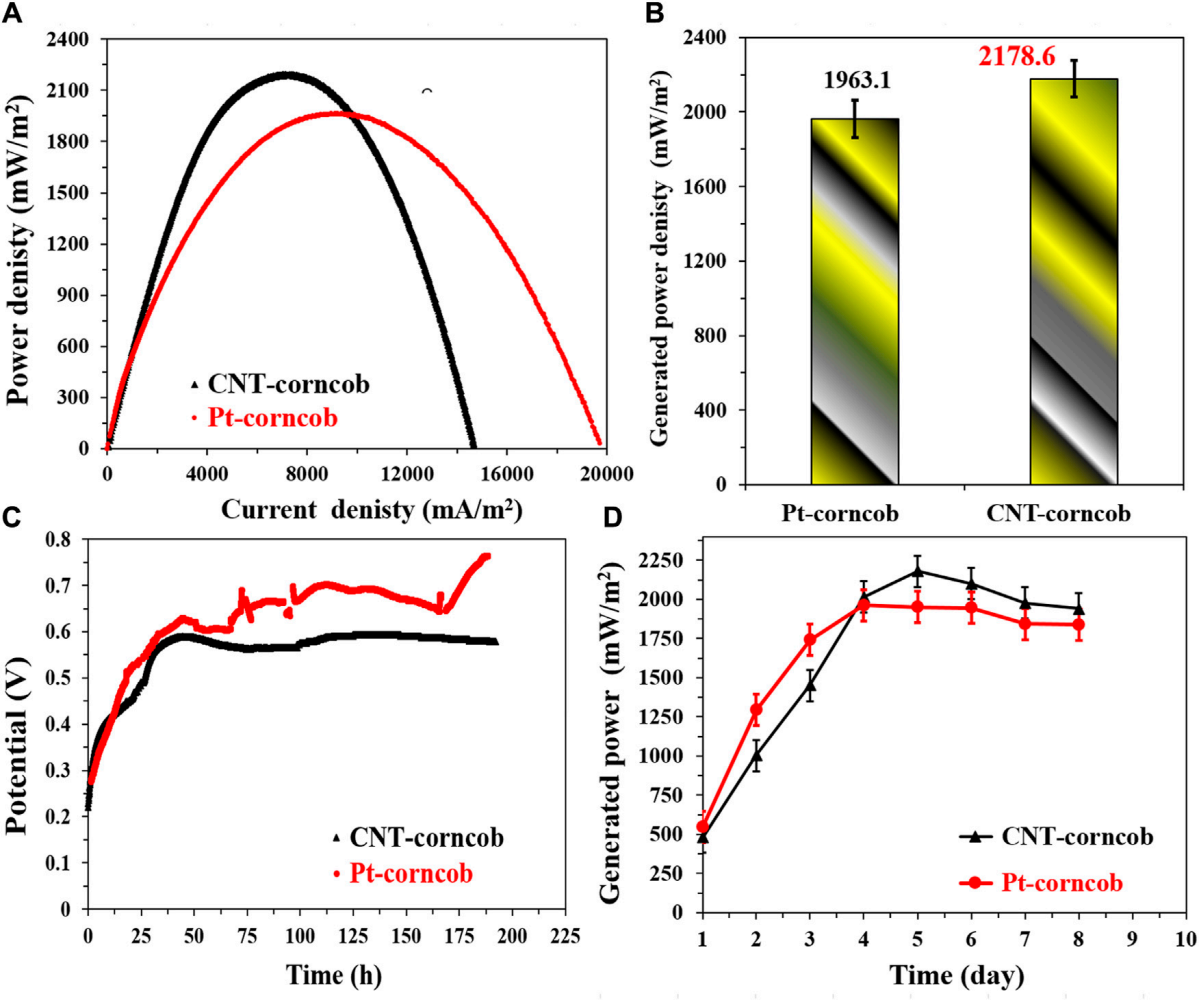
Performance of single chamber air cathodes MFCs driven by NaAc (5 g/L)/sewage wastewater and assembled from the proposed corncob-1000 anode using two different cathodes; standard Pt/C and the prepared CNTs/AC treated at 350°C and containing 0.035 g CNTs. **(A)** Polarization curves of the assembled microbial fuel cells, **(B)** Maximum generated power densities using the investigate cathodes, **(C)** the change of the open cell potential with respect with the time, and **(D)** Influence of running time on the generated powers from the assembled MFCs.

The comparison between the proposed CNTs/AC cathode and the standard Pt/C-loaded carbon cloth cathode in corncob-1000°C-based sewage wastewater-driven MFCs provides valuable insights into the performance of these cathode materials. The polarization curves shown in Figure 12A indicate that the CNTs/AC cathode-based MFC exhibits higher generated power densities compared to the Pt/C-based MFC. This result highlights the remarkable catalytic activity of the CNTs/AC cathode in promoting the oxygen reduction reaction (ORR), which is essential for the overall performance of microbial fuel cells (MFCs). The numerical data in Figure 12B further support this observation, with the CNTs/AC cathode-based MFC achieving a maximum generated power density of 2,178.6 ± 35 mW/m^2^, outperforming the Pt/C-based MFC with a power density of 1963.1 ± 32 mW/m^2^.

Notably, when examining the maximum current density obtained from the polarization curves, it is evident that the Pt/C-based MFC exhibits a higher current density compared to the CNTs/AC-based MFC. This difference in current density could be attributed to the higher conductivity of the Pt/C catalyst, which facilitates a more rapid electron transfer during the ORR process. However, it is important to note that the generated power density takes into account both the current density and the cell voltage, offering a comprehensive measure of the overall performance of the MFC.

The OCV variations displayed in Figure 12C indicate that the CNTs/AC cathode-based MFC achieves a highly stable open circuit voltage (OCV) over the course of the experiment, suggesting effective cathode performance and minimal losses in electrochemical potential. On the other hand, the Pt/C-based MFC shows a slightly higher maximum OCV but exhibits more fluctuations, implying that the CNTs/AC cathode provides better stability over time. The difference in the OCV behavior could be attributed to various factors, including the catalytic activity of the cathode materials, their interaction with the electrolyte, and the kinetics of the electrochemical reactions.

Figure 12D illustrates the maximum current density variation with time for both MFCs. Both cells follow a similar trend, with an initial period of gradual power increase in the first 4 days. However, the CNTs/AC-based MFC shows a more pronounced increase in power on the fifth day, followed by a gradual decrease until the end of the experiment. This behavior could be linked to the adaptation of the microorganisms to the cathode surface and the availability of organic compounds in the sewage wastewater. The relatively steady power output of the Pt/C-based MFC after the initial increase and the subsequent small decrease might indicate a more consistent microbial activity and substrate availability during the experimental period. In general, the comparison between the CNTs/AC cathode and the Pt/C-loaded carbon cloth cathode in case of utilizing corncob-driven anode highlights the excellent catalytic performance of the CNTs/AC material, resulting in higher generated power densities and greater stability in microbial fuel cells.

**FIGURE 13:**
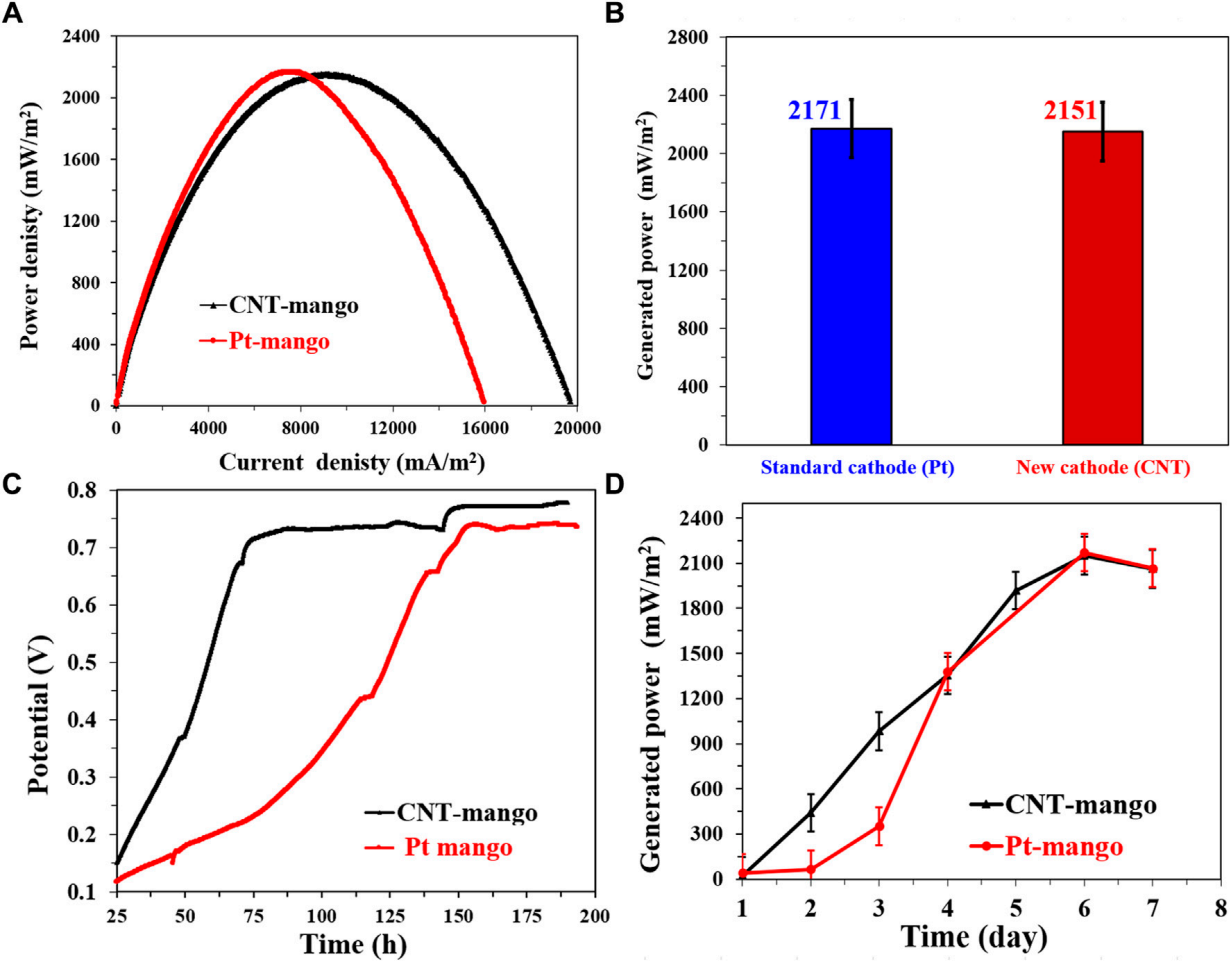
Performance of single chamber air cathodes MFCs driven by NaAc (5 g/L)/sewage wastewater and assembled from the proposed mango seed-1100 using two different cathodes; standard Pt/C and the prepared CNTs/AC treated at 350°C and containing 0.035 g CNTs. **(A)** Polarization curves of the assembled microbial fuel cells, **(B)** Maximum generated power densities using the investigate cathodes, **(C)** the change of the open cell potential with respect with the time, and **(D)** Influence of running time on the generated powers from the assembled MFCs.

The investigation of the performance of the proposed CNTs/AC cathode in conjunction with a mango seeds-based anode (prepared at 1,100°C) reveals interesting insights into the interplay between cathode and anode materials in microbial fuel cells (MFCs). Despite the similar power densities observed between the Pt/C and CNTs/AC cathode-based MFCs (2,151 and 2,171 mW/m^2^, respectively), there are notable differences in terms of current density, OCV behavior, and anode effect. Comparing the current densities obtained from the polarization curves (Figure 13A), it is evident that the CNTs/AC-based MFC achieves a higher maximum current density than the Pt/C-based MFC. This observation suggests that the CNTs/AC cathode material has a superior capability to facilitate electron transfer, leading to enhanced electrocatalytic activity during the oxygen reduction reaction (ORR) in case of utilizing the mango seed-based anode compared to the corncob one. Considering that the mango seed-based MFC generates higher maximum current density compared to the corncob-based cells (Figure 8), which can be attributed to the difference in the surface morphology of the two anodes (Figure 2), the higher current density in case of utilizing CNTs/Ac cathode indicates efficient utilization of the electrochemically active surface area of the cathode material in receiving excess electrons.

Shifting the focus to the OCV variations over time (Figure 13C), it is notable that the utilization of a mango seeds-based anode introduces a different trend compared to the corncob-based anodes. When Pt/C cathode is used with the mango seeds-based anode, a longer stabilization time for the OCV is required (157 h) compared to the CNTs/AC cathode (62 h). This difference can be attributed to variations in the interaction between the different anode materials, microbial community, and cathode materials. The higher OCV values observed for both anodes in comparison to the corncob-based anodes (Figure 12C) could be attributed to the unique surface characteristics of mango seeds and their potential to provide better attachment sites for microorganisms and electron transfer processes.

Lastly, the maximum power density variation with time (Figure 13D) shows that both the Pt/C and CNTs/AC cathode-based MFCs reached their peak power densities after 6 days of operation. Notably, the CNTs/AC-based MFC exhibits a faster initial increase in power density during the first 2 days compared to the Pt/C-based MFC. This behavior may result from the superior electrocatalytic activity of the CNTs/AC cathode, which promotes efficient ORR reactions, faster establishment of electron transfer pathways, and microbial adaptation on the cathode surface.

Overall, the results of the mango seeds-based anode with the proposed CNTs/AC cathode demonstrate the dynamic interplay between anode and cathode materials in MFCs. Despite the similar overall power densities, differences in current density, OCV behavior, and initial power increase suggest that the CNTs/AC cathode offers distinct advantages in terms of electron transfer and catalytic activity, resulting in efficient power generation and stability. The unique characteristics of the mango seeds-based anode further contribute to the overall performance by influencing microbial attachment and electron transfer dynamics.

### 3.5 COD removal in continuous mode MFCs

**FIGURE 14:**
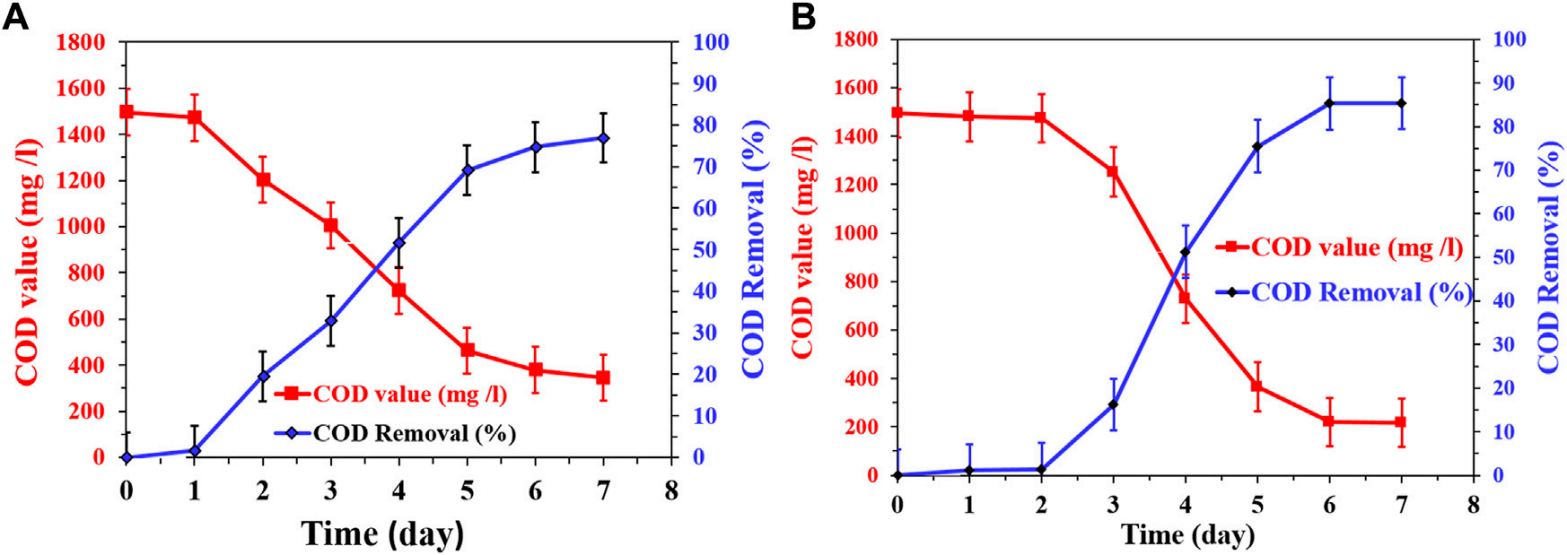
Change of COD removal using continuous mode MFCs at a feed flow rate 2 L/h using the proposed CNTs (7 wt%)/AC treated at 350°C with corncob-1000°C; **(A)** and mango seeds-1,100°C; **(B)** anodes.

The investigation of COD removal rates in continuous mode microbial fuel cells (MFCs), at a feed low rate of 2 L/h, using the newly proposed CNTs/AC cathode with corncob-1000°C and mango seed-1100°C anodes provides valuable insights into the efficiency of this system in terms of wastewater treatment. As depicted in Figure 14, the COD removal rate trends are distinct for the two different anode materials. In the case of the corncob-based MFC (Figure 14A), the COD removal rate shows an initial rapid increase from the first day until the fifth day, followed by a gradual decrease. This pattern suggests a robust start of microbial activities and organic matter degradation in the wastewater during the early operational phase of the MFC. However, as the process continues, factors such as substrate availability, microbial dynamics, and possibly accumulation of metabolic byproducts might contribute to the reduction in the removal rate. The observed maximum COD removal rate of 77% by the seventh day indicates that the system reaches a state of equilibrium between organic matter degradation and microbial activity.

Contrasting this, in the mango seeds-based MFC (Figure 14B), the COD removal rate shows a different behavior. Initially, there is minimal change in the COD content during the first 2 days, indicating a slower start in organic matter degradation compared to the corncob-based MFC. However, from the third day onwards, a rapid increase in the removal rate is observed until a peak removal rate of 85% is achieved by the sixth day, which remains stable on the seventh day. The enhanced COD removal rate in the mango seeds-based MFC compared to the corncob-based MFC could be attributed to the higher current density observed with the mango seeds-based anode. This higher current density suggests more efficient microbial activity, which in turn leads to improved degradation of organic compounds and enhanced COD removal. Additionally, the specific surface characteristics of the mango seeds-based anode could provide better attachment sites for microorganisms, thereby facilitating improved microbial growth and activity.

In summary, the observed differences in COD removal rates between the corncob and mango seeds-based MFCs highlight the intricate relationship between anode materials, microbial activity, and wastewater treatment efficiency. The higher current density associated with the mango seeds-based anode likely contributes to the accelerated COD removal rate observed in the mango seeds-based MFC. These findings underscore the importance of selecting appropriate anode materials to optimize microbial activity and wastewater treatment performance in continuous mode MFCs.

## 4 Conclusion

The graphitization temperature of biomass-derived anode materials played a crucial role in shaping their electrochemical characteristics. The higher graphitization temperatures led to improved electrical conductivity and enhanced microbial activity, resulting in higher power densities. The optimized graphitization temperatures of 1,000°C for corncob and 1,100°C for mango seeds anodes proved to be effective for achieving maximum power generation and stability. The incorporation of CNTs into the cathode composition proved highly effective in enhancing the ORR performance. The power density of the CNTs/AC cathode was significantly higher compared to commercial cathodes, demonstrating the superior electrocatalytic activity of the composite cathode. The influence of CNTs content in the cathode composition was investigated, revealing an optimal CNTs content (7 wt% with respect to the activated carbon) that yielded the highest power density. Moreover, thermal treatment of the prepared cathodes was explored, and it was found that a treatment temperature of 350°C yielded the best performance in terms of power density and stability. Comparative studies with platinum-loaded carbon cloth cathodes confirmed the favorable performance of the proposed CNTs/AC cathodes in terms of power density, current density, and stability. Additionally, the influence of different anode materials, such as corncob and mango seeds, was investigated. While both anodes showed promising results, mango seeds-based MFCs exhibited higher current densities and improved COD removal rates, underlining the importance of anode characteristics in MFC performance. Overall, the obtained results contribute to advancing the understanding of electrode materials’ impact on MFC performance and offer insights into the design of more efficient and sustainable energy conversion and wastewater treatment systems.

## Data Availability

The original contributions presented in the study are included in the article/Supplementary Material, further inquiries can be directed to the corresponding authors.
