# Supplementary material for: Synergistic advancements in sewage-driven microbial fuel cells: novel carbon nanotube cathodes and biomass-derived anodes for efficient renewable energy generation and wastewater treatment
Source: Front Chem. 2023 Nov 24;11:1286572. doi: 10.3389/fchem.2023.1286572 (PMC10704469; doi:10.3389/fchem.2023.1286572)
Supplement: Supplementary file 1 [file DataSheet1.PDF]

Synergistic Advancements in Sewage-driven Microbial Fuel Cells: Novel Carbon Nanotube Cathodes and Biomass-derived Anodes for Efficient Renewable Energy Generation and Wastewater Treatment

Nasser A. M. Barakat[1,*], Shimaa gamal[1], Hak Yong Kim[2,3], Nasser M Abd El-Salam[4,*], Hassan Fouad[5], Olfat A.Fadali[1], Hager M. Moustafa[1] and Omnia H. Abdelraheem[6]

[1]Chemical Engineering Department, Faculty of Engineering, Minia University, El-Minia 61516, Egypt.

[2]Department of Nano Convergence Engineering, Jeonbuk National University, Jeonju 54896, South Korea.

[3]Department of Organic Materials and Fiber Engineering, Jeonbuk National University, Jeonju 54896, South Korea.

[4]Natural Science Department, Community College, King Saud University, Riyadh 12642, Saudi Arabia.

[5]Biomedical Engineering Dept. Faculty of Engineering, Helwan University, Helwan, Egypt.

[6]Sciences Engineering Department, Faculty of Engineering, Beni-Suef University Beni-Suef 62511, Egypt.

Corresponding authors:

Nasser A. M. Barakat

Tel: +20862348005, Fax: +20862364420

E-mail: *nasbarakat@mu.edu.eg*

Nasser M Abd El-Salam

E-mail: nelsalam@ksu.edu.sa

Sewage water characterization

In the study of sewage water characterization, the utilization of advanced instruments and techniques is crucial for obtaining precise and comprehensive data. Samples were collected from the drainage system and subjected to comprehensive characterization using advanced instruments at the laboratories of the Sanitation and Drinking Water Company in El-Minya, Egypt. The characterization process involves the analysis of various parameters, ranging from physical and chemical properties to microbiological aspects. Here are some of the advanced instruments and techniques employed in sewage water characterization:

1. High-Performance Liquid Chromatography (HPLC): HPLC is a powerful technique for the quantification of organic compounds such as volatile fatty acids, phenols, and other organic matter in sewage water. It allows for high-resolution separation and quantification of individual components, providing valuable insights into water quality.
2. Gas Chromatography-Mass Spectrometry (GC-MS): GC-MS is utilized for the identification and quantification of volatile organic compounds (VOCs) in sewage water. This technique is particularly useful in detecting contaminants, such as pesticides and pharmaceuticals, which may not be adequately removed by conventional wastewater treatment processes.
3. Inductively Coupled Plasma-Mass Spectrometry (ICP-MS): ICP-MS is employed to determine trace metal concentrations in sewage water. It offers exceptional sensitivity, making it suitable for detecting heavy metals and metalloids, which can have detrimental effects on the environment and human health.
4. Atomic Absorption Spectroscopy (AAS): AAS is a precise method for quantifying specific metals in sewage water. It is commonly used to measure elements like lead, cadmium, and chromium, which are often present in wastewater due to industrial and domestic discharges.
5. Next-Generation Sequencing (NGS): In the context of microbial community analysis, NGS is invaluable for identifying and quantifying microorganisms present in sewage water. It enables researchers to gain insights into the diversity and abundance of bacterial and archaeal species in microbial fuel cells (MFCs) fed with sewage water.

6. Electrochemical Sensors: These sensors are utilized for in-situ measurements of parameters such as pH, dissolved oxygen, and redox potential. They provide real-time data to monitor the electrochemical behavior of MFCs and assess their performance.
7. Titration Methods: Titration is used for the determination of parameters like alkalinity and acidity in sewage water. It provides insights into the water's buffering capacity and potential for pH fluctuations.

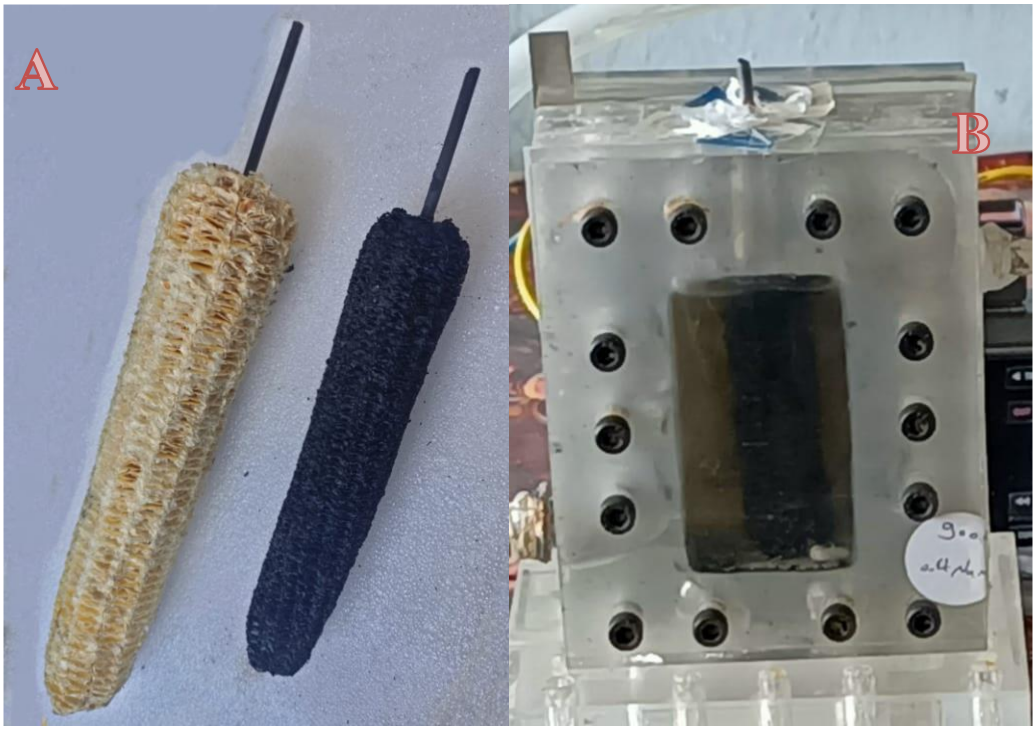

Fig. S1 Photo images for the corncob before and after the carbonization process; (A), and anode chamber assembly; (B).

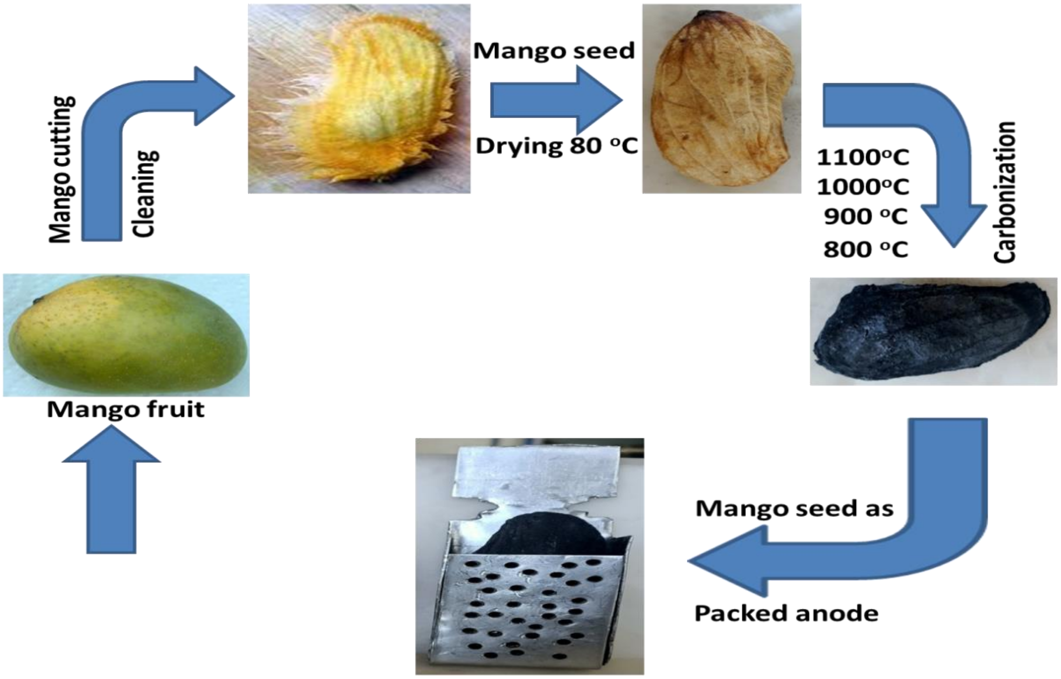

Figure S2: The preparation procedure of anode packed structure from mango fruit.

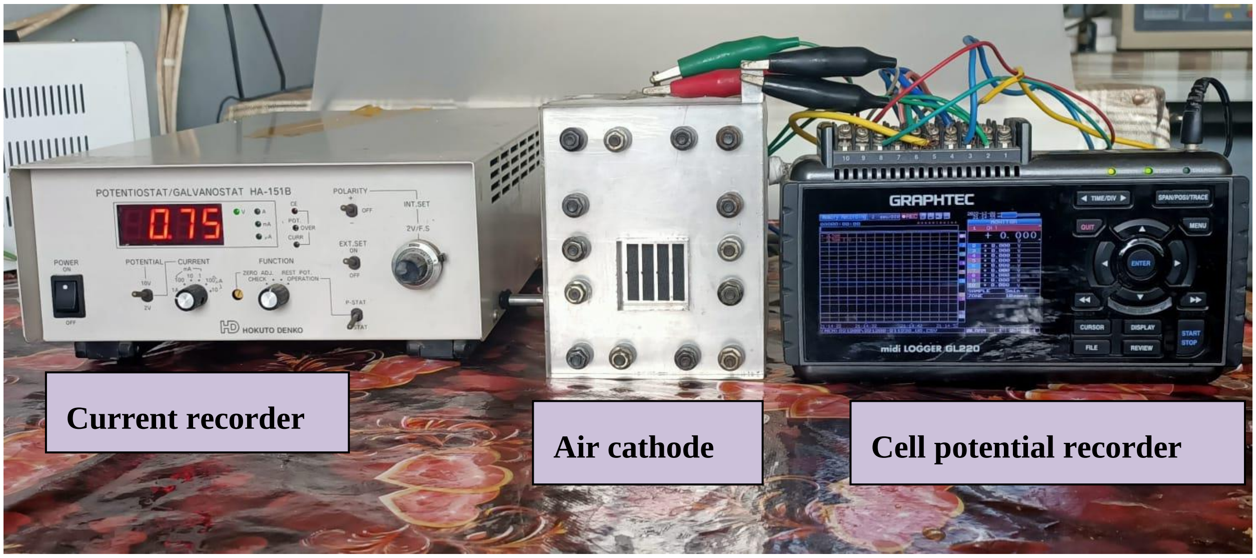

Fig. S3 Photo image for the batch mode assembled air-cathode microbial fuel cell using the municipal wastewater.

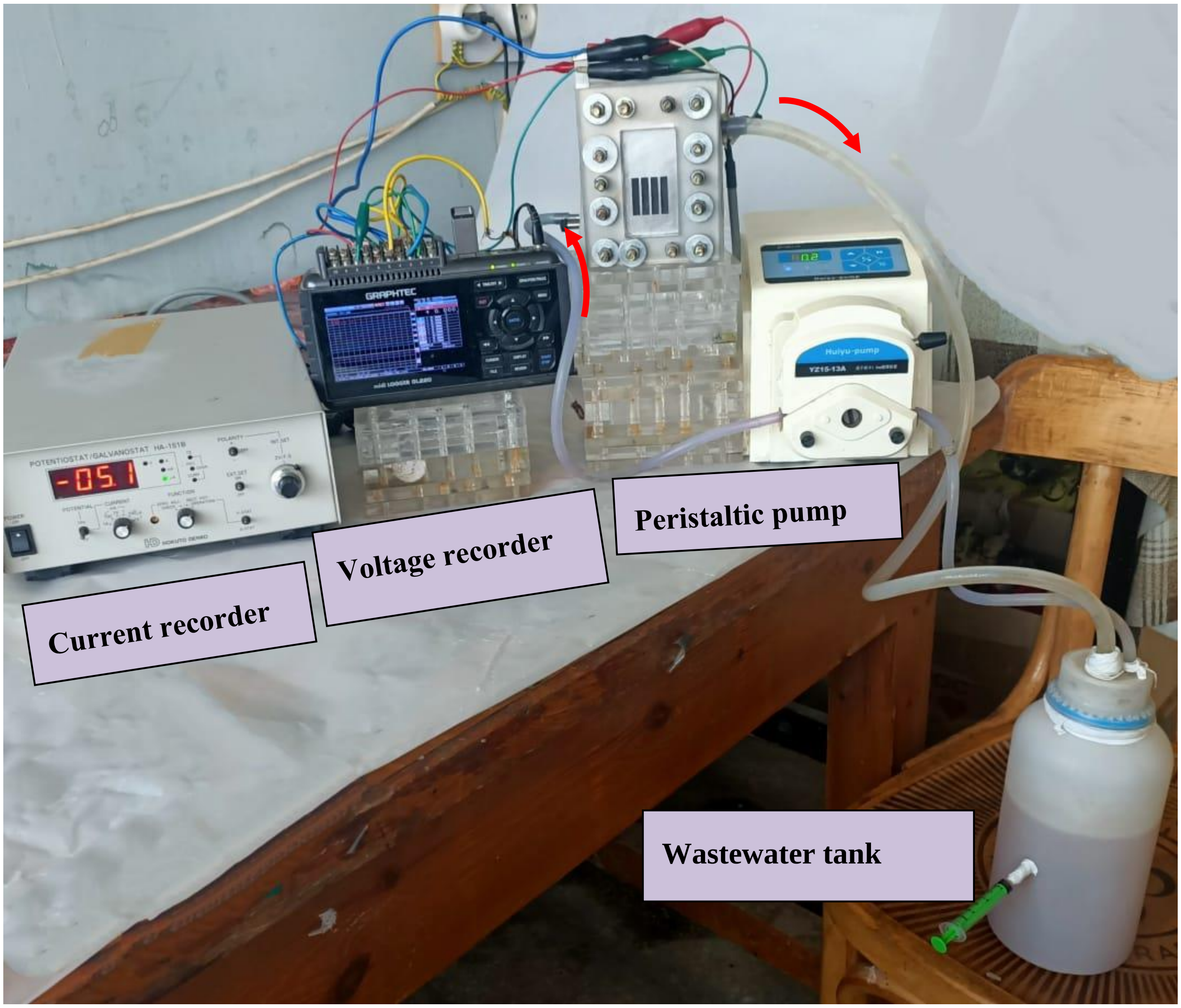

Fig. S4 Photo image for the continuous mode air-cathode microbial fuel cell using the municipal wastewater. The red arrows denote to the solution direction.
